# hnRNP A1 and hnRNP F Modulate the Alternative Splicing of Exon 11 of the Insulin Receptor Gene

**DOI:** 10.1371/journal.pone.0027869

**Published:** 2011-11-23

**Authors:** Indrani Talukdar, Supriya Sen, Rodolfo Urbano, James Thompson, John R. Yates, Nicholas J. G. Webster

**Affiliations:** 1 VA San Diego Healthcare System, San Diego, California, United States of America; 2 Department of Medicine and the Biomedical Sciences Graduate Program, University of California San Diego, La Jolla, California, United States of America; 3 The Scripps Research Institute, Department of Cell Biology, La Jolla, California, United States of America; National Institutes of Health - National Cancer Institute, United States of America

## Abstract

Exon 11 of the insulin receptor gene (*INSR*) is alternatively spliced in a developmentally and tissue-specific manner. Linker scanning mutations in a 5′ GA-rich enhancer in intron 10 identified AGGGA sequences that are important for enhancer function. Using RNA-affinity purification and mass spectrometry, we identified hnRNP F and hnRNP A1 binding to these AGGGA sites and also to similar motifs at the 3′ end of the intron. The hnRNPs have opposite functional effects with hnRNP F promoting and hnRNP A1 inhibiting exon 11 inclusion, and deletion of the GA-rich elements eliminates both effects. We also observed specific binding of hnRNP A1 to the 5′ splice site of intron 11. The SR protein SRSF1 (SF2/ASF) co-purified on the GA-rich enhancer and, interestingly, also competes with hnRNP A1 for binding to the splice site. A point mutation -3U→C decreases hnRNP A1 binding, increases SRSF1 binding and renders the exon constitutive. Lastly, our data point to a functional interaction between hnRNP F and SRSF1 as a mutant that eliminates SRSF1 binding to exon 11, or a SRSF1 knockdown, which prevents the stimulatory effect of hnRNP F over expression.

## Introduction

The insulin receptor (IR) is encoded by a single gene (*INSR*), which is composed of 22 exons [1]. Among these 22 exons, the 36 nt long exon 11 of the *INSR* gene is alternatively spliced to give two IR isoforms; IR-A which excludes exon 11 and IR-B which includes exon 11. The relative expression of IR isoforms depends on species, tissue-type, developmental stage and pathological condition. While IR-A is widely expressed, IR-B has more limited distribution, being expressed predominantly in insulin-sensitive tissues, such as liver, muscle, adipocytes and kidney, suggesting a metabolic role [2], [3], [4].

Splicing of pre-mRNA involves the excision of introns in the primary gene transcript and ligation of the exons to form the mRNA. This process occurs by the recognition of specific sequences at the exon-intron boundaries by small nuclear ribonucleoproteins or snRNPs [5], [6]. Inclusion of individual exons can be enhanced or silenced by the binding of specific splicing factors to the primary RNA transcript. A family of serine-arginine rich proteins (SR proteins) plays a crucial role in alternative splicing of mRNA by binding to exonic and intronic sites to enhance exon recognition and interacting with components of the U1 and U2 snRNPs to facilitate their binding to the 5′ and 3′ splice sites [7], [8], [9], [10], [11]. Indeed we have previously published that SRSF3 (SRp20) and SRSF1 (SF2/ASF) bind to sites within exon 11 of the *INSR* gene to promote exon inclusion [12].

The heterogeneous nuclear ribonucleoproteins (hnRNPs) are a family of related proteins that lack an SR domain [13]. All hnRNP family proteins share structural homology with amino-terminal RNA binding domains (RRMs or KHs) and a carboxy-terminal glycine rich domain and bind to RNA in a cooperative manner. The hnRNP-A/B subfamily acts predominantly as repressors, but the hnRNP F/H subfamily can act as activators as well as repressors [14]. One potential mechanism by which hnRNPs inhibit splicing involves competition for RNA binding sites with SR proteins [14], [15], [16], [17] thus interfering with the recruitment of snRNPs, but hnRNP proteins can inhibit splicing by additional mechanisms [18], [19]. Cooperative binding of hnRNPs to high-affinity sites can also contribute to silencing by recruiting molecules of hnRNP to lower affinity binding sites and displacing SR proteins [20]. Cooperative binding may also explain how some hnRNPs promote splicing by multimerizing across introns and bringing the consecutive exons in close proximity thus looping out very large introns. This phenomenon allows U1 and U2 snRNPs to interact across large introns which eventually facilitate the inclusion of the alternatively spliced exon [21].

We previously demonstrated exonic and intronic regulatory elements in the insulin receptor gene that regulate alternative splicing of exon 11. Two SR proteins, SRSF3 and SRSF1, bind to exonic splicing enhancer elements to promote exon inclusion and CELF1 (CUG-BP1), a CELF-family protein, inhibits exon inclusion by binding to both exonic and intronic silencer elements [12]. In this paper we identify hnRNP F and -A1 as additional proteins that bind to intronic and exonic splicing regulatory elements to antagonistically regulate the alternative splicing of exon 11. hnRNP F binds to both ends of intron 10 resulting inclusion of exon 11. hnRNP A1 binds similarly to intron 10 but also binds to the 5′splice site of intron 11 resulting in repression of exon 11 inclusion. Moreover, the relative expression of hnRNP F and A1 correlates with splicing of the endogenous *INSR* gene in HepG2 and HEK293 cells.

## Results

### Consensus binding sites for hnRNP A1, -F/H are found at the 5′ GA-rich sequence in intron 10 of IR

**Figure 1:**
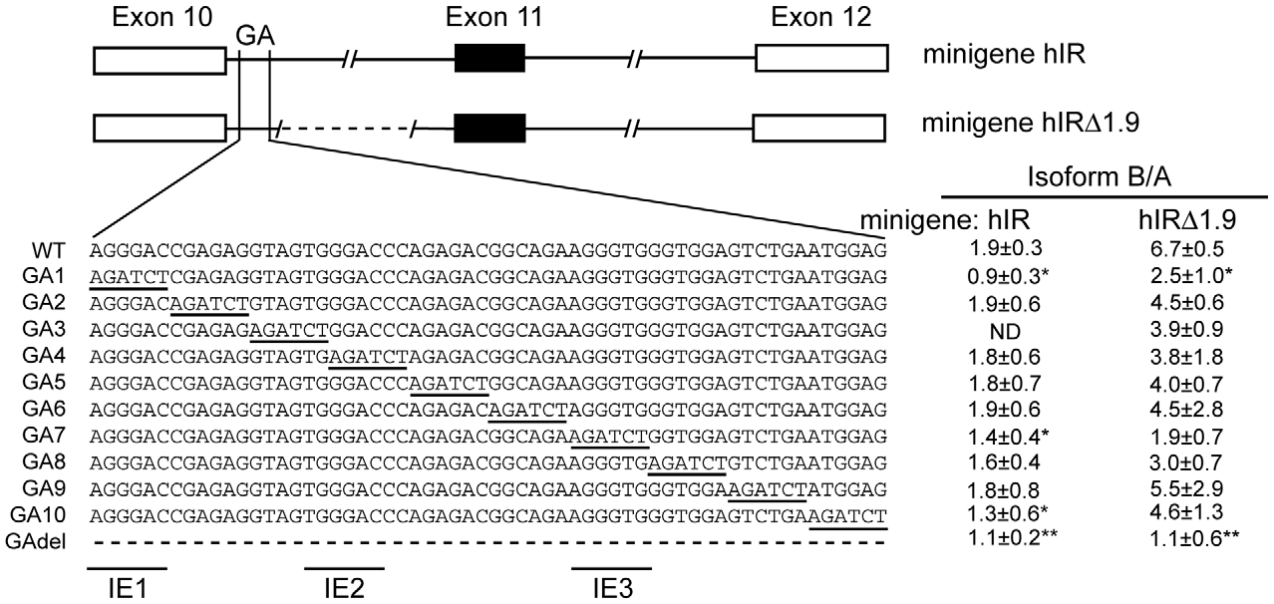
A GA-rich sequence is located near the 5′ region of intron 10 of *INSR*. A schematic diagram of minigenes hIR and hIRΔ1.9 is shown. White boxes represent exon 10 and 12 and the black box represents exon 11. The intervening lines indicate the introns in between. Dotted lines indicate deletions. Solid perpendicular lines in minigene hIR and hIRΔ1.9 demonstrate the boundary of the GA rich sequence. Sequences of GA rich region and the series of linker scanning mutants (GA1-10) are shown below. The ratio of isoform IR-B that includes exon 11 to isoform IR-A that excludes exon 11 (± standard deviation) is given at the right hand site for minigene hIRΔ1.9 and hIR as well as the mutants, after transient transfection into HepG2 cells. Asterisks indicate statistical significance (*p≤0.05, ** p≤0.01) vs. wild-type sequence for n = 4 experiments. ND represents not determined. IE1, IE2 and IE3 indicate the intron elements used for subsequent in vitro binding experiments.

A sixty nucleotide GA-rich element is located near the 5′ end of intron 10 of IR gene and deletion of this region decreases the inclusion of exon 11 in an IR minigene [22] suggesting a potential intronic enhancer element. A series of linker scanning mutants (GA1-10) was created by inserting a BglII restriction site in minigene hIR, which contains the entire 2.3 kb intron 10, to localize this enhancer element. The same linker scanning mutations were made in minigene hIRΔ1.9 containing an internal deletion of 1.9 kb in intron 10. We have previously shown that this deletion increase exon incorporation [22]. A number of the mutants show decreased incorporation of exon 11 suggesting that multiple regulatory elements within the GA-rich region contribute to the enhancer function (Figure 1). Mutants GA1, GA7 and GA10 decreased exon 11 inclusion significantly in minigene hIR and mutants GA1 and GA7 decrease exon 11 inclusion in minigene hIRΔ1.9. Comparison of the sequences in linker scanning mutants GA1, 7 and 10 identified a common AGGGA motif. A similar motif UGGGA is also found spanning mutants GA3 and 4 but neither substitution altered exon inclusion significantly, possibly as each substitution was only a partial mutation of the site. The triple G elements identified by mutants GA1, 4 and 7 were labeled IE1 (intronic element 1), IE2 and IE3, respectively, for subsequent *in vitro* binding analysis.

### hnRNP A1 and -F/H and SRSF1 bind to the G-rich elements in intron 10

**Figure 2:**
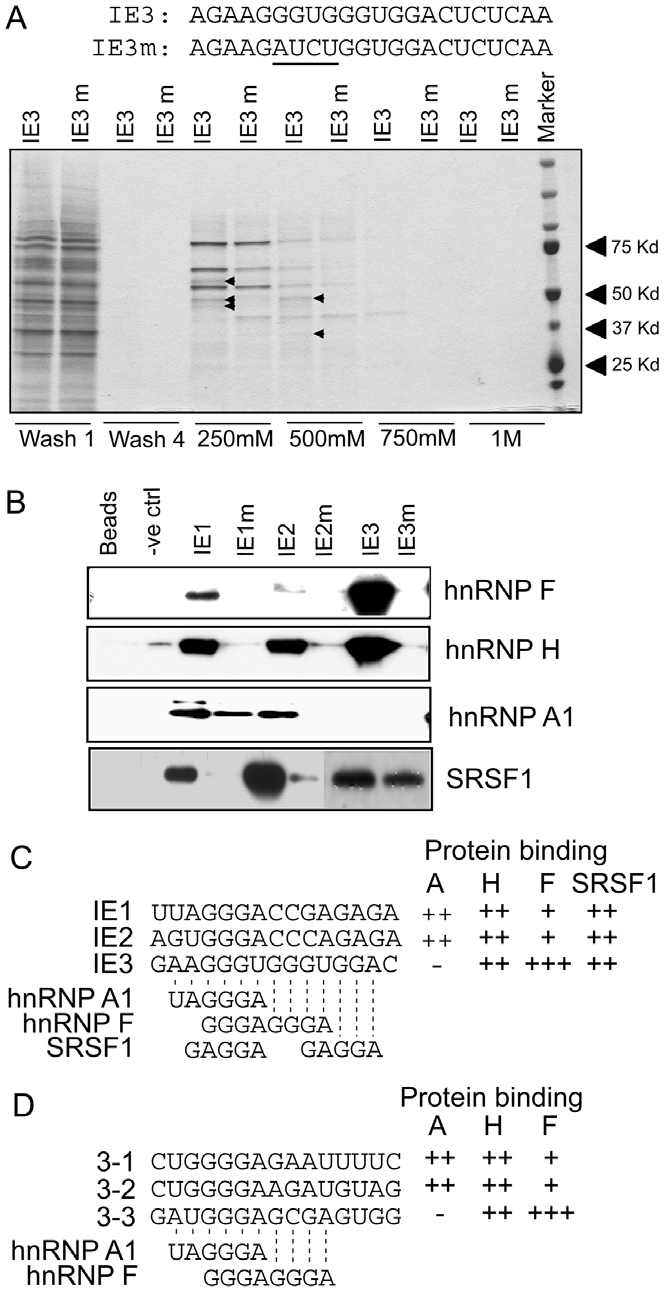
hnRNP A1 and -F/H and SRSF1 bind to G-rich motifs in intron 10. Panel A: RNA-affinity purification of HeLa nuclear proteins using templates IE3 or IE3m, which contains the GA7 mutation. Sequences of IE3 and IE3m are given at top. Bound proteins were washed four times with 100 mM KCl and then eluted with increasing concentrations of KCl. Proteins from washes 1 and 4 and from the KCl elution were separated on a 4–12% Bis-Tris gel and stained with Coomassie blue. Arrowheads indicate differential protein bands between the two RNA templates. Panel B: In vitro RNA pull-down assay using RNA templates IE1, IE2 and IE3 and their cognate mutants. Bound proteins were separated by gel electrophoresis and immunoblotted for hnRNP A1, hnRNP H, hnRNP F or SRSF1. Panel C: Summary of binding to 5′ GA-rich motifs. The consensus binding sites for hnRNP F [34], hnRNP A1 [35], and SRSF1 [36] are shown. Relative binding associated with the RNA is indicated by plus signs. Minus signs denote no detectable binding. Panel D: Summary of binding to 3′ GA-rich motifs. The consensus binding sites for hnRNP F and hnRNP A1 are shown. Relative binding associated with the RNA is indicated by plus signs. Minus signs denote no detectable binding.

To identify proteins that recognize the repeated AGGGA motif, we analyzed nuclear proteins that bind to IE3 and its mutant IE3m (GA7). RNA oligos spanning the sequences IE3 and IE3m were synthesized and coupled to adipic-acid dihydrazide beads. These RNA templates were used to affinity purify proteins from HeLa nuclear extracts. We used HeLa extracts rather than HepG2 extracts as the former can support *in vitro* splicing of the IR mRNA. Proteins were eluted with increasing concentrations of KCl and the affinity-selected proteins were characterized by Mudpit mass spectrometry (Figure 2A). The most abundant proteins showing specific binding to IE3 were hnRNP F and hnRNP H1, followed by hnRNP H2 and hnRNP U (scaffold attachment factor A) (Table 1 and Material S1). We also identified other proteins that bound equally to IE3 and IE3m including NONO/SFPQ, SRSF1, PTBP1, hnRNP L and hnRNP A1/A2. NONO/SFPQ forms a heterodimer that binds polypyrimidine tracts and interacts with PTBP1 to promote spliceosomal assembly [23], [24], [25]. SRSF1 is a SR protein that promotes alternative exon usage. This is intriguing as we have already published that SRSF1 promotes exon 11 inclusion by binding to an exonic enhancer [12]. To validate the binding of the hnRNP proteins and SRSF1 to the predicted sequences, an *in vitro* RNA pull-down assay was performed using synthetic RNAs spanning IE1, IE2, IE3 and the IE1m (GA1), IE2m (GA4) and IE3m (GA7) mutants. The pull down assay was performed with Hela nuclear extract as before then followed by western blot analysis using antibodies to hnRNP A1, F, and H and SRSF1 (Figure 2B). Greater hnRNP F binding was observed with IE3, than with IE1 and IE2, as this site contains closely spaced GGG triplets, which have been shown to bind to RRM1 and RRM2 of hnRNP F [26]. In contrast, hnRNP A1 bound to IE1 and IE2 but only weakly to IE3. Lastly, hnRNP H and SRSF1 bound consistently to all templates. The pull down assay was repeated with recombinant hnRNP A1 and hnRNP F with similar results (data not shown). Summary of the results and alignments to conserved hnRNP A1, hnRNP F and SRSF1 binding sites are indicated in Figure 2C. Inspection of intron 10 for additional GA-rich sites revealed three such sites at the 3′ end of the intron. RNAs containing these sites (3-1, 3-2, 3-3) were also used in the RNA-affinity pull-down assay and they were found to bind hnRNP A1, hnRNP H and hnRNP F similar to the sites at the 5′ end of the intron. The binding data are summarized in Figure 2D.

**Table 1:** Splicing factors bound to IE3 and its mutant by Mudpit analysis.

Splicing Factors	Calculated mass (Da)	Spectrum count	
		IE3	IE3mut
Specific Binding			
hnRNP H1	49229	355	140
hnRNP F	45672	162	93
hnRNP H2	49264	80	31
hnRNP U	90480	69	18
hnRNP H3	36926	22	0
Non-Specific Binding			
NONO	54232	663	621
SFPQ	76150	424	526
SRSF1	27745	109	89
hnRNP A2/B1	37430	53	66
PTBP1	57221	42	60
hnRNP A1	38846	25	25
hnRNP L	60187	17	23

### Over expression or knockdown of hnRNP A1 and hnRNP F modulates inclusion of exon 11 of an IR minigene

**Figure 3:**
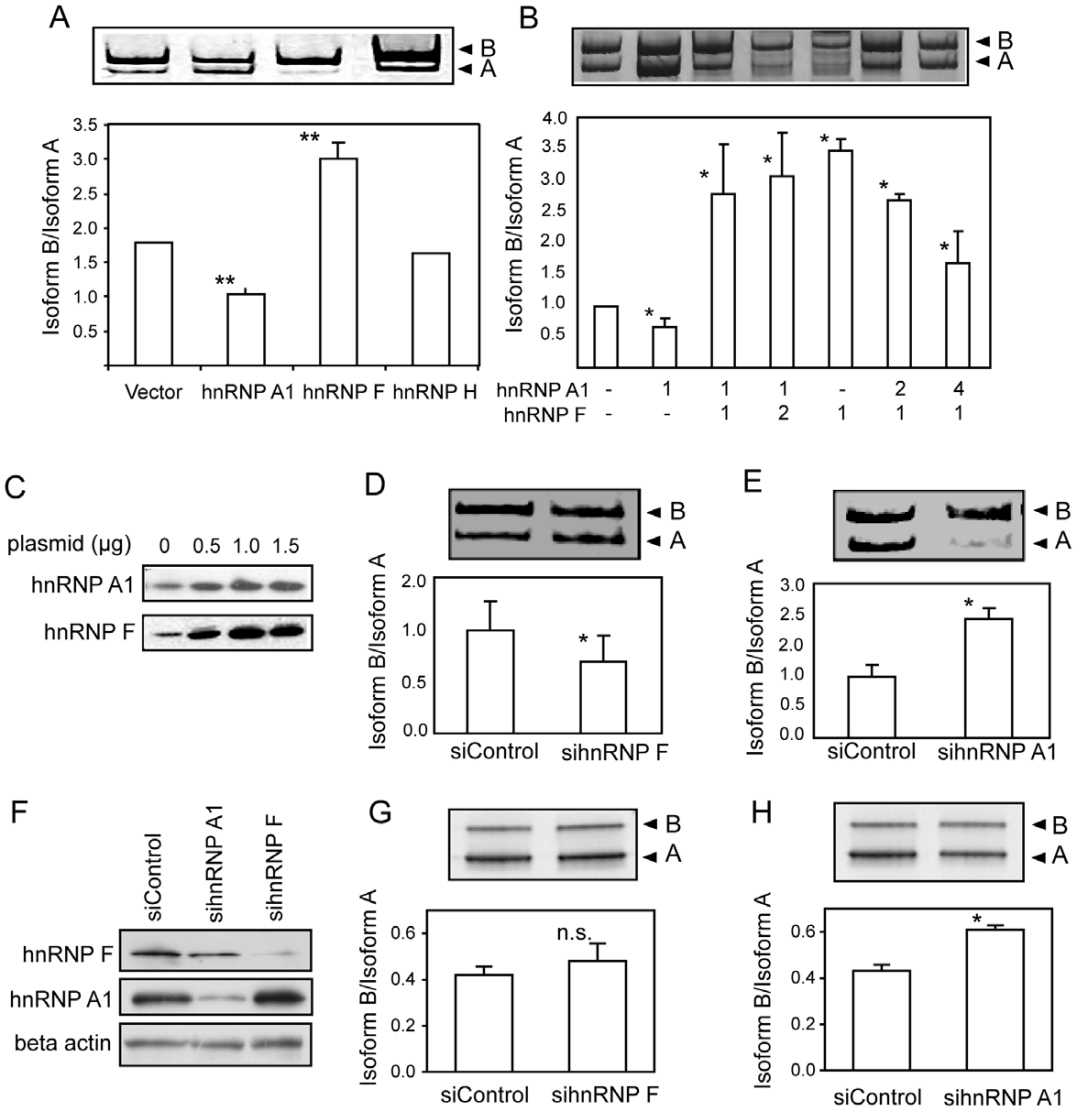
Over expression of hnRNP A1 decreases and hnRNP F increases inclusion of exon 11 of an *INSR* minigene, whereas knockdown of endogenous hnRNP F and hnRNP A1 has the opposite effect. Panel A: Transient transfection of minigene hIR with the expression vectors for hnRNP A1, F or H was performed in HepG2 cells. The relative expression level of the IR isoforms was measured by RT-PCR analysis. For the control, minigene hIR was transfected with the empty expression vector. The graph indicates the ratio isoform B/isoform A normalized to the value of minigene hIR alone and the values are mean ± SD for n = 5 experiments. A representative gel is shown. Asterisks indicate significant changes in exon inclusion compared to the minigene hIR transfection alone (** p≤ 0.01). Panel B: Antagonistic effects of hnRNP A1 and F. Minigene hIR was co-transfected with vector alone or with increasing amounts of hnRNP A1 or F (µg) keeping the amount of the other factor constant. The graph indicates the ratio of isoform B/isoform A ± standard deviation, and the values are normalized to the value of minigene hIR alone for n = 3 experiments. Asterisks indicate statistical significance vs. vector alone (* p≤ 0.05). Panel C: Over-expression of hnRNP A1 and hnRNP F was tested by transient transfection of 0.5, 1 and 1.5 µg of the respective expression plasmids, followed by immuno-blot analysis. The increase in expression level was compared with the non-transfected endogenous level. Panel D: siRNA against hnRNP F was co-transfected with minigene hIR in HepG2 cells. Panel E: siRNA against hnRNP A1 was co-transfected with minigene hIR in HepG2 cells. Representative gels are shown. The graphs show the quantification of isoform B/isoform A normalized to minigene hIR in the presence of the control siRNA (mean ± SD, n = 3). Asterisk indicates statistical significance vs. control siRNA (*p≤0.05). Panel F: Knockdown of hnRNP F and hnRNP A1 following siRNA transfection into HepG2 cells. Panel G: Effect of hnRNP F knockdown on *INSR* gene splicing. Panel H: Effect of hnRNP A1 knockdown on *INSR* splicing. Representative gels are shown. The graphs show the quantification of isoform B/isoform A (mean ± SD, n = 3).

To test whether hnRNP A1, -H, or -F can regulate exon 11 inclusion, we co-transfected HepG2 cells with the hIR minigene and expression vectors for hnRNP A1, -F or -H. Analysis of exon 11 splicing of the minigene construct indicated that over expression of hnRNP A1 significantly decreases and hnRNP F significantly increases the inclusion of exon 11 (Figure 3A). Over-expression of hnRNP H does not have any effect on exon 11 inclusion in HepG2 cells (Figure 3A). As hnRNP A1 and -F appeared to play opposite roles in splicing of exon 11 despite binding to similar sequences, we titrated the hnRNP A1 and hnRNP F expression plasmids on the IR minigene. Transfection of increasing amounts of hnRNP F expression plasmid in face of a constant quantity of hnRNP A1 causes an increase in the inclusion of exon 11, compared to hnRNP A1 alone (Figure 3B). Conversely, transfecting increasing amounts of hnRNP A1 expression plasmid in the face of a constant quantity of hnRNP F causes a dose-dependent decrease in the inclusion of exon 11 compared to hnRNP F alone (Figure 3B). We verified that hnRNP A1 and hnRNP F were over-expressed by immunoblotting (Figure 3C). Taken together, these data suggest that hnRNP F and hnRNP A1 not only bind at multiple sites of intron 10, but also play opposite roles in regulating exon 11 inclusion. This may provide an explanation for the relatively small effect due to some linker scanning mutations such as GA4 as binding of both hnRNP A1 and hnRNP F is lost.

To further confirm the role of endogenous hnRNP F and -A1 on alternative splicing of exon 11, we used RNA interference to knockdown the endogenous proteins. As expected, co-transfection of the hIR minigene with siRNAs against hnRNP A1 and hnRNP F shows the opposite effect on splicing compared to the over-expression experiments above. siRNA against hnRNP F significantly decreases inclusion of exon 11 compared to the control siRNA (Figure 3D) and co-transfection of siRNA against hnRNP A1 significantly increases the inclusion of exon 11 (Figure 3E). We then investigated the effect of the knockdown on the endogenous *INSR* gene. The siRNAs reduced the level of hnRNP A1 or hnRNP F proteins by immunoblotting (Figure 3F). The siRNA against hnRNP F did not alter the splicing of the endogenous *INSR* gene (Figure 3G) but knockdown of hnRNP A1 significantly increased exon 11 incorporation in agreement with the minigene result (Figure 3H). These results confirmed the ability of hnRNP A1 to repress exon incorporation in both an artificial IR minigene and also more importantly the endogenous *INSR* gene. The lack of an effect of hnRNP F knockdown on the endogenous gene may indicate that hnRNP F does not bind the endogenous RNA transcript or may reflect a functional redundancy that is observed on the endogenous gene that is absent on the simplified minigene. Indeed, while the minigene mimics much of the regulation of the endogenous gene, exon 11 incorporation is significantly less for the endogenous gene perhaps suggesting additional negative regulatory factors.

### Linker scanning mutants spanning of individual binding sites do not disrupt the effects of hnRNP A1 and hnRNP F

**Figure 4:**
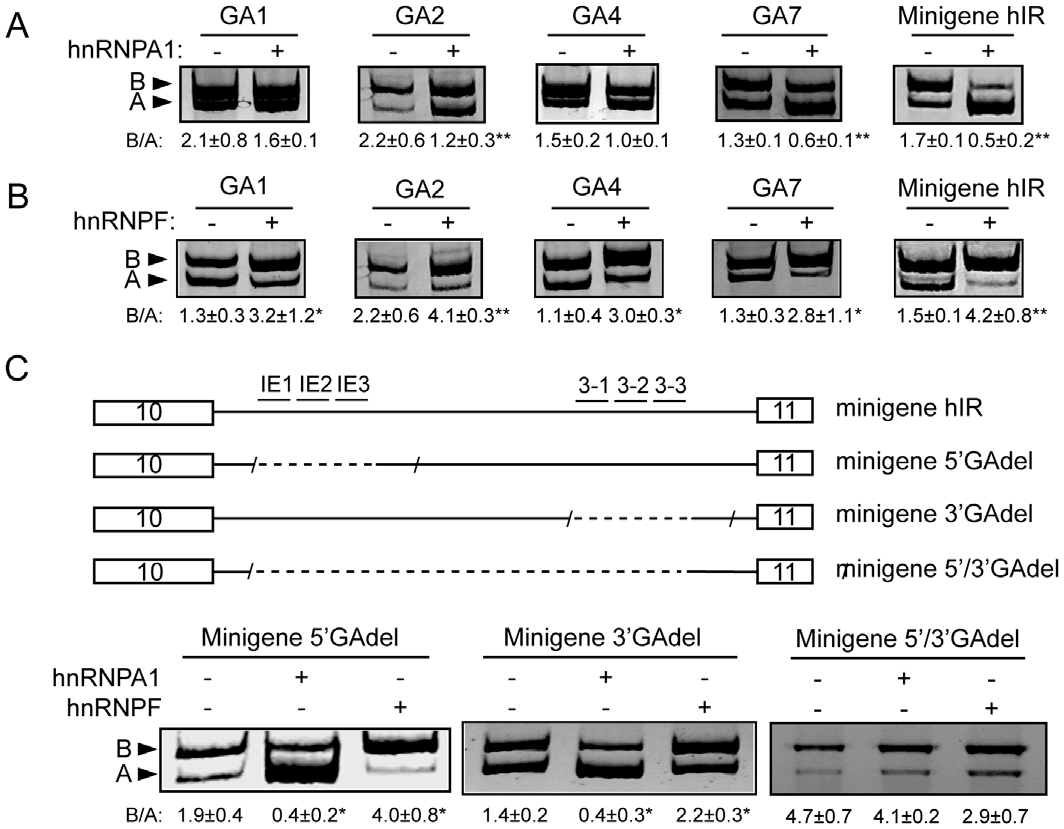
Linker scanning mutants do not disrupt the effect of hnRNP A1 or hnRNP F. Panels A and B: Co-transfection of linker scanning mutants GA1, 2, 4 and 7 and minigene hIR with the expression vector for hnRNP A1 (Panel A) and hnRNP F (Panel B). Panel C: Schematic of the deletion constructs spanning 5′ (IE1, IE2 & IE3) or 3′ (3-1, 3-2 & 3-3) binding sites (5′GAdel and 3′GAdel, respectively), or the combined 5′/3′GA deletion construct, which lacks all GA-rich binding sites and the central 1.9 kb of the intron. Deletion mutants were co-transfected with hnRNP A1 or hnRNP F in HepG2 cells. Total RNA was isolated 48h after co-transfection. The relative expression level of the IR isoforms was measured and analyzed as described previously. The ratio of isoform B over isoform A of n = 3 experiments with ± SD is shown. Asterisks indicate statistical significance vs. vector alone (* p≤0.05, **p≤0.01).

As hnRNP A1 and hnRNP F bind to multiple sites within the GA-rich enhancer in intron 10, we tested whether mutation of individual binding sites would disrupt the effect of either protein on exon 11 inclusion. Linker scanning mutants GA1, GA2, GA4 and GA7 were transfected with the expression vectors for hnRNP A1 or -F (Figure 4A and 4B). Mutants GA1 and GA4 responded partially to hnRNP A1, but GA7, where hnRNP A1 binds poorly, and control mutant GA2, respond by decreasing exon 11 inclusion similar to minigene hIR. Similar effects were seen with the GA mutants in minigene hIRΔ1.9 (data not shown). In contrast, hnRNP F increased exon 11 inclusion on all mutants comparable to minigene hIR. These observations suggested redundant effects through binding to multiple sites. To eliminate this redundancy, we tested the deletion construct that lacks all the GA-rich binding sites at the 5′ end of the intron (minigene 5′GAdel). Transfection of this deletion mutant into HepG2 cells did not eliminate the ability of hnRNP A1 to repress and hnRNP F to enhance exon 11 incorporation (Figure 4C). Since the linker scanning and deletion mutations at the 5′ end did not abolish the effect of hnRNP A1 and F, we looked at the other potential binding sites for these proteins at the 3′ end of intron 10. We deleted a 203 nucleotide segment of intron 10 that contains the 3′ GA-rich elements (minigene 3′GAdel). The deletion starts 95 nucleotides upstream of exon 11 so does not interfere with the branch-point sequence or 3′ splice site. As seen for the 5′ sites, removal of the 3′ binding sites did not eliminate the effect of hnRNP A1 or hnRNP F on exon 11 incorporation (Figure 4C). Since neither the 5′ nor the 3′ deletions eliminated the effect of hnRNP A1 or -F, we made a deletion construct which eliminated both the 5′ and 3′ GA-rich binding sites and the 1.9 kb intervening sequence (minigene 5/3′GAdel), effectively eliminating all potential binding sites for hnRNP A1/F in intron 10. Transfection of this complete deletion mutant into HepG2 cells elevated exon 11 inclusion over the intact intron, similar to minigene hIRΔ1.9 (Figure 1), but more importantly eliminated the ability of both hnRNP A1 to decrease and hnRNP F to increase exon inclusion (Figure 4C).

### hnRNP A1 also binds at the junction of exon 11 and intron 11 to repress exon 11 inclusion

**Figure 5:**
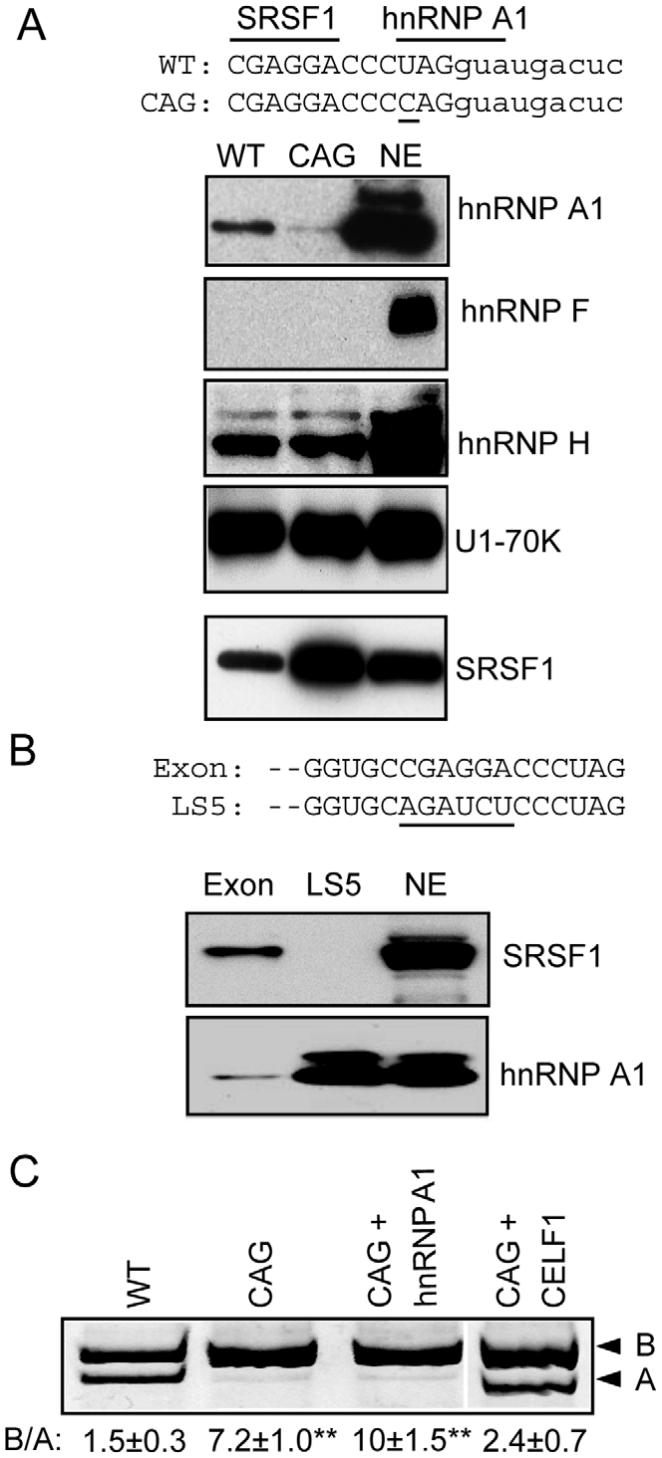
hnRNP A1 but not hnRNP F binds at the junction of exon 11 and intron 11 to repress exon inclusion. Panel A: The nucleotide sequence of the RNA templates of the wild type exon 11/intron 11 junction and the -3U→C mutation (underlined). Binding sites for SRSF1 and hnRNP A1 are indicated by bars above. Representative blots show the results of *in vitro* RNA pull-down assays followed by immunoblotting for hnRNP A1, F, H, and U1-70K and SRSF1. Panel B: The nucleotide sequence of the RNA templates of the wild type exon 11 and the LS5 mutation (underlined). Representative blots show the results of *in vitro* RNA pull-down assays followed by immunoblotting for hnRNP A1 and SRSF1. Panel C: Transient transfection of the wild type minigene hIR and the -3U→C mutant in the absence and presence of the expression vector of hnRNP A1 or CELF1. A representative gel is shown. The ratio of isoform B over isoform A of n = 3 experiments with ± SD is shown. Asterisks indicate statistical significance vs. wild type (** p≤ 0.01).

Since hnRNP A1 and hnRNP F bind similar sequences and have similar effects on splicing in other systems, we were curious why these two factors have opposite effects on the *INSR* gene. We noted that SRSF1 binds to the same intronic G-rich element as hnRNP F and -A1, and we had shown previously that SRSF1 binds exon 11 to promote inclusion [12], we wondered whether hnRNP A1 or -F could also bind to the exon. On closer examination of the SRSF1 binding site at the 3′ end of exon 11, we noticed that the adjacent exon 11-intron 11 junction (UAG|GUAAGA) resembles an hnRNP A1 binding motif (UAGGGA). We tested whether hnRNP F, A1 and H bind to this sequence using a synthetic RNA spanning this splice site in an in vitro binding assay. We found strong binding of hnRNP A1 and hnRNP H, but not hnRNP F, to this RNA (Figure 5A). To disrupt the potential hnRNP A1 binding site without altering the complementarity to the U1 snRNA, we substituted the -3U with C (CAG mutant). Mutation of U to C at this position decreases binding of hnRNP A1 but not hnRNP H or U1-70K. We also noticed that binding of SRSF1 to this sequence increases with the CAG mutation, which suggests steric competition between hnRNP A1 and SRSF1 for binding to adjacent sites. The competition was verified by measuring hnRNP A1 binding to exon 11 or to a mutant template (LS5) containing a linker scanning mutation in the SRSF1 binding site (CGAGGA → AGATCT). hnRNP A1 binding is greatly increased by the LS5 mutation (Figure 5B). Transfection of the minigene carrying the splice site CAG mutation drastically increases exon 11 incorporation and eliminates the effect of hnRNP A1 to repress exon inclusion in HepG2 cells (Figure 5C). In contrast CELF1 (CUG-BP1), another negative regulator of *INSR* alternative splicing [12], still represses exon 11 inclusion in the CAG mutant. This finding suggests the inhibitory role for hnRNP A1 is due to binding at this splice site rather than in the upstream intron.

### Mutation of the SRSF1 binding site on exon 11 disrupts the effect of hnRNP F on *INSR* splicing

**Figure 6:**
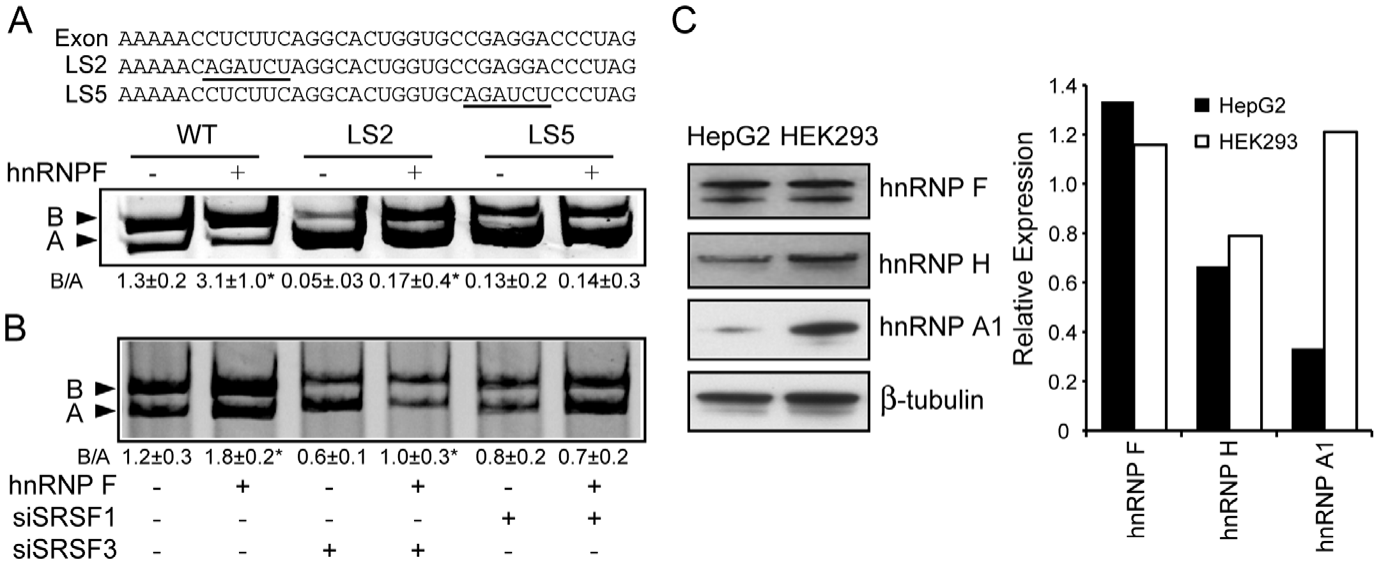
Functional dependence of hnRNP F on SRSF1. Panel A: Co-transfection of wild-type and the linker scanning mutants LS2 or LS5 with or without expression plasmid of hnRNP F. Sequence of the wild-type exon and the LS2 and LS5 mutants are shown underlined. Panel B: Co-transfection analysis in HepG2 cells with or without the over expression of hnRNP F in the presence of siRNA against endogenous SRSF3(SRp20) or SRSF1(SF2/ASF). Total RNA was isolated 48h after co-transfection. The relative expression level of the IR isoforms was measured and analyzed as described previously. The ratio of isoform B over isoform A of n = 3 experiments with ± standard deviation is shown. Asterisks indicate statistical significance vs. vector alone (* p≤ 0.05, **p≤ 0.01). Panel C: Total protein was extracted from HepG2 and HEK293 cells. Equal amounts of protein were loaded and immunoblotted for hnRNP A1, F, H, or β-tubulin as a loading control. Graph shows relative expression normalized to β-tubulin.

As SRSF1 binding in intron 10 overlaps with hnRNP A1, -F and -H binding, we wanted to test whether disruption of the exonic SRSF1 binding site modulates the effect of hnRNPs on *INSR* splicing. Surprisingly, mutation LS5, which eliminates the ability of SRSF1 to promote exon inclusion [12], also eliminates the ability of hnRNP F to increase exon 11 inclusion, whereas disruption of the exonic SRSF3 binding site (LS2: CTCTTC → AGATCT) has no effect (Fig. 6A). To confirm that hnRNP F depends on SRSF1, we co-transfected cells with the expression vector of hnRNP F along with siRNA against SRSF1 or against SRSF3 as a control. The effect of hnRNP F to increase exon 11 incorporation is abolished in the SRSF1 knock-down cells (Figure 6B) but not in the SRSF3 knock-down cells (Figure 6B). These results suggest a functional interaction between hnRNP F and SRSF1, but we were unable to detect physical interaction between these two proteins by co-immunoprecipitation or by *in vitro* pull-down assay with recombinant His-tagged hnRNP F (data not shown).

### hnRNP A1 is differentially expressed in HepG2 and HEK293 cell lines and correlates with skipping of exon 11

To assess whether alterations of hnRNP A1 or -F might underlie differential alternative splicing in HepG2 and HEK293 cells, we measured endogenous splicing factor expression in these two cells. As we have published previously, exon 11 inclusion is predominant in HepG2 hepatoma cells (>70% inclusion) whereas exon 11 is almost completely skipped (<10% inclusion) in HEK293 embryonic kidney cells [12]. Although hnRNP F and -H levels are similar in these two cells, hnRNP A1 is expressed at a much higher level in HEK293 cells (Figure 6C). Consistent with this observation, knockdown of hnRNP A1 in HEK293 cells causes a small increase in exon 11 incorporation (B/A ratio 0.21±0.04 for si-hnRNP A1 vs. 0.16±0.09 for si-ctrl, data not shown).

## Discussion

We previously showed that alternative splicing of exon 11 of IR gene is mediated through cis-acting sequences within intron 10 and we identified a GA-rich intronic splicing enhancer at the 5′ end of intron 10. In this paper we localized the enhancer elements within this region to a repetitive AGGGA motif using linker-scanning mutagenesis. *In vitro* RNA binding assays and mass spectrometry revealed that these G-rich motifs are binding sites for the hnRNP proteins hnRNP A1, hnRNP H and hnRNP F, and the SR protein SRSF1. The finding of SRSF1 binding to the upstream intron is intriguing given our previous finding of SRSF1 binding to an exonic enhancer element to promote exon 11 inclusion. Co-transfection and knock-down studies indicated that hnRNP A1 suppresses but hnRNP F enhances exon 11 incorporation. hnRNP H does not have any effect on IR minigene splicing in HepG2 cells, which was somewhat surprising as hnRNP H has been shown to repress exon 11 splicing in conjunction with CELF1 in muscle cells [27]. This may be related to the observation that CELF1 is not expressed at a high level in HepG2 cells. Furthermore we have found that hnRNP A1 protein is expressed at a much higher level in HEK293 cells, where exon 11 is almost completely excluded, than HepG2 cells where exon 11 is mostly incorporated. This suggests that the relative ratio of hnRNP A1 and -F may play a role in determining the extent of exon 11 inclusion. hnRNP A1 is not be the only negative regulator of exon 11 inclusion in HEK293 cells however as knockdown only causes a small increase in exon inclusion. These cells express high levels of CELF1 that also represses exon inclusion, so the function of hnRNP A1 and CELF1 to cause exon skipping may be redundant (12). Mutation of the individual binding sites does not disrupt the effects of hnRNP A1 and hnRNP F on exon 11 inclusion suggesting either multiple redundant binding sites or alternative binding sites elsewhere in the gene. Indeed, we found that these proteins also bind to the 3′ end of intron 10 but deletion of neither the 5′ sites nor the 3′ sites by themselves could abolish the effect of hnRNP A1 or hnRNP F. When all intronic binding sites were eliminated, however, neither hnRNP A1 nor hnRNP F modulated exon 11 inclusion suggesting that binding of these proteins at both ends of intron is required for their functional effect on *INSR* splicing.

Surprisingly, we also found that hnRNP A1, but not hnRNP F, binds strongly to the exon 11-intron 11 junction. This suggested an explanation for the functional difference between hnRNP A1 and hnRNP F, in that binding of hnRNP A1 could impair recognition of this splice site leading to exon skipping. The sequence of the exon 11-intron 11 boundary of *INSR* gene is UAG|GUAUGA (the consensus 5′ splice site is MAG|GURRGU, M is A or C; R is purine). This splice site resembles an hnRNP A1 binding motif (UAGGGA). A search of the SpliceRack database shows that -3U is only found in 2.5% of 5′ splice sites (as opposed to 23% for the -3C) suggesting evolutionary pressure to avoid this nucleotide so that the splicing junction does not resemble an hnRNP A1 binding site. We showed that a single U→C mutation at the -3 position disrupts the hnRNP A1 binding site homology without interrupting complementarity to the U1 snRNA, decreases hnRNP A1 binding, increases SRSF1 binding, and eliminates the effect of hnRNP A1 on *INSR* splicing. The increase in SRSF1 binding and the decrease in hnRNP A1 binding, suggests steric competition for the exonic binding site between SRSF1 and hnRNP A1 to antagonize *INSR* splicing. Interestingly, the elimination of the exonic binding site for SRSF1 results in the complete disruption of the hnRNP F effect, even though intronic hnRNP F and SRSF1 binding sites remain intact. This requirement was confirmed as knock down of SRSF1, but not SRSF3, eliminated the effect of hnRNP F over expression. More importantly, we showed that the -3 U→C mutation renders the exon constitutive. This observation has important implications for alternative splicing. It is generally thought that the splice sites associated with alternatively spliced exons are weak and diverge from the consensus [28], [29], [30], [31], [32]. Much of this evidence is based on mutational analysis, where increasing complementarity to the U1 snRNA increases splice site usage. However, splice site prediction algorithms are not always consistent with observed results. For example, the splice site score for exon 11 is comparable with other constitutive exons in *INSR*. Predictions of splice site strength using Markov and positional weight matrix models for splice site scores indicate that exons 4-8 and exon 19 of *INSR* bear similar if not weaker splice sites, yet only exon 11 is subject to alternative splicing. Our data suggest that binding of a negative regulatory protein such as hnRNP A1 at a splice junction can modulate the splice site strength and cause skipping of the exon even if the splice junction sequence is predicted to be strong. From the SpliceRack database, we identified 75 genes that contain an exact match to exon 11 splice site sequence (UAGGUAUGA). Interestingly, 64 of them (85%) are alternatively spliced using the AStalavista (Alternative Splicing transcriptional landscape visualization tool) web server. In contrast, we identified 614 genes that have an exact match to the -3C exon 11 sequence (CAGGUAUGA) and 471 of them (77%) are alternatively spliced. This suggests that -3 U splice sites are found in genes undergoing alternative splicing at a greater frequency, with the caveat that we do not know whether these exons themselves are the site of alternative splicing. Our results suggest a model in which the interaction between hnRNP A1 proteins across intron 10 and the exon 11-intron 11 boundary causes skipping of exon 11. It would be interesting to see whether a similar mechanism of alternative splicing occurs in those 64 genes that contain the exact UAGGUAUGA sequence at the splicing junction and further studies are planned to test the generality of this model.

**Figure 7:**
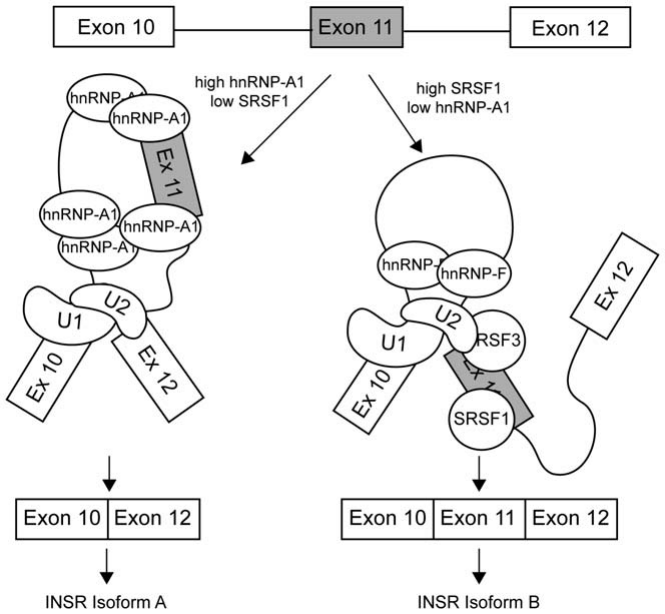
Model for antagonistic regulation of exon 11 inclusion by hnRNP A1 and hnRNP F/SRSF1. When hnRNP A1 is highly expressed compared to SRSF1, binding of hnRNP A1 to the 5′ splice site of intron 11 and the intronic elements blocks recognition of the exon by the splicing apparatus causing skipping of the exon. When hnRNP A1 is low compared to SRSF1, binding of SRSF1 to the exon prevents binding of hnRNP A1 allowing binding of SRSF3 and hnRNP F that promote exon inclusion.

The overall mechanism of the regulation of *INSR* splicing by various factors is complex. Binding of hnRNP A1 and hnRNP F to both ends of the 2.3 kb intron 10 might be expected to increase exon 11 inclusion, as Martinez-Contreras et al. have shown that hnRNP binding to sites at both ends of an intron brings the two ends together via protein-protein interactions, thus looping out the intervening sequence and facilitating splicing [21]. The finding that deletion of either the 5′ or 3′ binding sites does not abrogate the ability of hnRNP F to increase exon inclusion, however, argues against the looping model. One possible model is that the steric competition between SRSF1 and hnRNP A1 at the exon 11-intron 11 junction determines whether the exon is recognized by the splicing apparatus (Figure 7). If hnRNP A1 levels are high, then hnRNP A1 binds to the splice site and intronic sequences thus blocking both the 5′ and 3′ splice sites on exon 11. Indeed, Zhu et al. have shown that hnRNP A1 binding is propagated along RNA through co-operative binding interactions from an initial high-affinity binding site [20]. In contrast if SRSF1 is high and binds to the exon, hnRNP A1 is prevented from coating the exon allowing other factors such as hnRNP F and SRSF3 to bind to promote exon recognition. This would explain the dependence of hnRNP F stimulation on the SRSF1 binding site in exon 11. Whether this model holds for our system will require further investigation.

## Materials and Methods

### Immobilization of RNA on agarose beads and RNA binding assays

Synthetic RNAs were purchased from Integrated DNA Technologies Inc (Coralville, IA). RNA affinity purification was performed by modification of a published procedure [33]. HeLa cell nuclear extract was purchased from Accurate Chemical and Scientific Corporation (Westbury, NY). Briefly, 1000 pmol of RNA was oxidized with sodium *m*-periodate and covalently coupled to 400 µl of 50% slurry of adipic acid dihydrazide-agarose beads (Sigma, St. Louis, MO). The beads were washed three times with 2 M NaCl and then equilibrated with buffer D (20 mM HEPES-KOH, pH 7.6, 10% (v/v) glycerol, 150 mM KCL, 0.2 mM EDTA and 0.5 mM dithiothreitol) with 2.5mM ATP. RNA-agarose bead slurry was incubated with 75 µl of HeLa nuclear extract under splicing conditions at 30°C for 25 minutes in buffer D in a total volume of 600 µl. The beads were washed five times with buffer D then bound proteins were eluted by boiling in 60 µl of 2X protein sample buffer. The affinity-selected proteins were separated by electrophoresis on a 4–12% Bis-Tris gel (Bio-Rad Laboratories, Hercules, CA) and analyzed by Western blotting. Sequences of RNA templates are as follows: IE1-UUAGGGACCGAGAGG, IE1m-UCACCACCCGAGAGG, IE2-GUAGUGGGACCCAGAGA, IE2m- GUACACGCACCCAGCGA, IE3-AGAAGGGUGGGUGGACUCUCAA, IE3m-AGAAGAUCUGGUGGACUCUCAA, IE4-CUGGGGAAGAUGUAGCUCACUCCGUCUAGCAAGUGAGGGAGCCAGUGG, WT- CGAGGACCCUAGGUAUGACUCACCUGUGCG, CAG- CGAGGACCCCAGGUAUGACUCACCUGUGCG. The sequence of the exon 11, LS2 and LS5 templates are as published [12].

### Sample preparation and Mass-spectrometry analysis

RNA affinity purification with the IE3 and the IE3m mutant (GA7) RNA oligos was performed as above. After incubation with Hela nuclear extract under splicing conditions, the RNA-agarose beads were washed and then bound proteins were eluted with increasing concentrations of KCl (250 mM, 500 mM, 750 mM and 1 M). The eluents were precipitated with 2 volumes of chilled acetone and centrifuged at 14,000 rpm for 10 minutes at 4°C. The pellet was washed and dried. The RNA affinity purified proteins were analyzed by 4–20% SDS-PAGE electrophoresis. The 500 mM fraction was analyzed by Mudpit, multidimensional chromatographic separation coupled with tandem MS/MS (Material S1).

### Plasmid constructs

The wild-type IR minigene (hIR) and a deleted mutant (hIRΔ1.9) have been described previously [22]. Minigene hIR contains the entire 2.3 kb intron 10 whereas minigene hIRΔ1.9 has a 1.9 kb deletion in the center of the intron. All other plasmids were constructed using standard techniques. Linker-scanning mutants were generated by PCR mutagenesis using the Quickchange mutagenesis kit (Stratagene, La Jolla, CA). A BglII restriction site was systematically inserted every six nucleotides throughout the GA-rich enhancer in intron 10. Plasmids expressing hnRNP F, hnRNP H and hnRNP A1 were purchased from ATCC (Manassas, VA).

### Cell culture, transfections and RNA extraction

HepG2, HeLa and HEK293 cells were obtained from A.T.C.C. (Manassas, VA) and maintained routinely in minimum essential medium plus. Earle's salts (Invitrogen, Carlsbad, CA) with 10% fetal bovine serum at 37°C under 10% CO_2_. Cells were plated at a density of ∼1×10^6^ cells/well in six-well plates. Medium was changed every 2 days. Transfections were performed with Fugene-6 (Roche, Indianapolis, IN) or Transfast (Promega, Madison, WI) transfection reagents according to the manufacturer's protocol. Cells were harvested 48 h after transfection and total cellular RNA was prepared using RNAzol B (Tel-Test Inc, Friendswood, TX).

### Reverse transcription and amplification of cDNA

Total RNA (1μg) was reverse transcribed using oligo-dT (Applied Biosystem, Foster City, CA). The endogenous and the transfected minigene IR transcripts were amplified using primer pairs as described previously [22] and reactions were visualized on 12% polyacrylamide gels after staining with ethidium bromide. The white and black images were inverted and the splicing products were quantified with Kodak Electrophoresis Documentation and Analysis System (EDAS) 290.

### siRNA transfections and analysis

Double-stranded, pre-annealed siRNA oligonucleotides against hnRNP A1, SRSF1, SRSF3 (Santa Cruz Biotechnology, Santa Cruz, CA), hnRNP F (Integrated DNA Technologies Inc) or a control siRNA (Santa Cruz) were co-transfected in HepG2 cells with the *INSR* minigene at 100 nM concentration with Fugene 6. To assess the effect of the knockdown on the endogenous *INSR* gene, hnRNP A1 and F ‘on target plus, smartpool’ siRNAs were purchased from Dharmacon and were tranfected into HepG2 cells using Lipofectamine 2000 (Invitrogen, CA), using the manufacturer's protocol. Forty-eight hours after transfection, cells were harvested and assayed for mRNA expression. Relative expression levels of isoform B and isoform A of the IR minigene were measured as described before.

### Whole cell lysate and western blot analysis

Whole cell lysates were prepared from HepG2 and HEK293 cells by using boiled lysis buffer (10 mM Tris, 1% SDS). Protein concentration was measured using DC protein assay kit (Bio-Rad). Equal amounts of protein were separated on 4–12% Bis-Tris gels and immunoblotted with antibodies against hnRNP A1, -F, -H, SRSF1 and beta-tubulin (Santa Cruz Biotechnology).

## Supporting Information

Material S1 **Mass spectrometry analysis following RNA affinity purification.** MudPIT Mass Spectrometry data for IE3 (Sample: wt1) starting on page 2 and for IE3 mutant (Sample: m1) on page 19. Columns show the protein symbol, protein description, number of peptide sequences, number of spectra, sequence coverage, and molecular weight of protein.
(PDF)
