# Supplementary material for: hnRNP A1 and hnRNP F Modulate the Alternative Splicing of Exon 11 of the Insulin Receptor Gene
Source: PLoS One. 2011 Nov 23;6(11):e27869. doi: 10.1371/journal.pone.0027869 (PMC3223206; doi:10.1371/journal.pone.0027869)
Supplement: Material S1 — Mass spectrometry analysis following RNA affinity purification. MudPIT Mass Spectrometry data for IE3 (Sample: wt1) starting on page 2 and for IE3 mutant (Sample: m1) on page 19. Columns show the protein symbol, protein description, number of peptide sequences, number of spectra, sequence coverage, and molecular weight of protein. (PDF) [file pone.0027869.s001.pdf]

Results from Mass Spectrometry Analysis of RNA Affinity Purification run by Dr. John Yates at the YRC at Scripps Research Institute, La Jolla CA.

MudPIT Mass Spectrometry data for IE3 (Sample: wt1) page 2

MudPIT Mass Spectrometry data for IE3 mutant (Sample: m1) page 19

View Mass Spectrometry Run

Run Information:

Organism:	*Homo sapiens*
Run Date:	2008-03-11
Project:	Mechanism of alternative splicing of human insulin receptor
DTA SELECT:	Download DTASelect Filter text<br>Download DTASelect HTML file<br>Download Unfiltered DTASelect text file
Comments:<br>[Edit Comments]	RNA binding splicing regulatory proteins sample: wt1

FILTERED Run Results: [unfilter]

	Hit Protein	Protein Desc	Sequence Count	Spectrum Count	Sequence Coverage	Mol. Wt.
[View Peptides]	RPLP2	ribosomal protein, large, P2	5	10	67.8%	11665
[View Peptides]	HNRPH3	heterogeneous nuclear ribonucleoprotein H3 (2H9)	11	22	55.8%	36926
[View Peptides]	NONO	non-POU domain containing, octamer-binding	62	663	54.8%	54232
[View Peptides]	HNRNPA2B1	heterogeneous nuclear ribonucleoprotein A2/B1	15	53	52.1%	37430
[View Peptides]	SEPT2	septin 2	17	75	50.4%	41487
[View Peptides]	GTF2I	general transcription factor II, i	62	358	47.0%	112416
[View Peptides]	HNRPH1	heterogeneous nuclear ribonucleoprotein H1 (H)	23	355	46.8%	49229
[View Peptides]	HNRPF	heterogeneous nuclear ribonucleoprotein F	18	162	45.8%	45672
[View Peptides]	SNRPD2	small nuclear ribonucleoprotein D2 polypeptide 16.5kDa	5	22	44.9%	13527
[View Peptides]	HNRPH2	heterogeneous nuclear ribonucleoprotein H2 (H')	19	80	43.9%	49264
[View Peptides]	SEPT9	septin 9	24	113	42.2%	65369
[View Peptides]	SEPT7	septin 7	15	66	42.1%	50680
[View Peptides]	PACSIN3	protein kinase C and casein kinase substrate in neurons 3	19	76	41.0%	48487
[View Peptides]	ACTB	actin, beta	19	111	40.8%	41737

[View Peptides]	SFRS1	splicing factor, arginine/serine-rich 1 (splicing factor 2, alternate splicing factor)	15	109	40.7%	27745
[View Peptides]	SUB1	SUB1 homolog (S. cerevisiae)	6	10	37.0%	14395
[View Peptides]	SFPQ	splicing factor proline/glutamine-rich (polypyrimidine tract binding protein associated)	51	424	36.2%	76150
[View Peptides]	GAPDH	glyceraldehyde-3-phosphate dehydrogenase	9	34	36.1%	36053
[View Peptides]	RPS8	ribosomal protein S8	6	12	35.6%	24205
[View Peptides]	PARP1	poly (ADP-ribose) polymerase family, member 1	37	131	34.8%	113084
[View Peptides]	DDX1	DEAD (Asp-Glu-Ala-Asp) box polypeptide 1	25	125	34.5%	82432
[View Peptides]	PTBP1	polypyrimidine tract binding protein 1	18	42	34.1%	57221
[View Peptides]	RPL30	ribosomal protein L30	3	6	33.9%	12784
[View Peptides]	KRT10	keratin 10 (epidermolytic hyperkeratosis; keratosis palmaris et plantaris)	15	45	33.6%	59519
[View Peptides]	gi|119594658, gi...	gi|8980669|gb|AAF82266.1|AF229068_1 HSPC170 protein [Homo sapiens], gi|7705477|ref|NP_057488.1| hypo...	4	5	32.8%	14199
[View Peptides]	PRKDC	protein kinase, DNA-activated, catalytic polypeptide	141	410	32.2%	469093
[View Peptides]	HNRNPR	heterogeneous nuclear ribonucleoprotein R	17	35	31.4%	70943
[View Peptides]	THOC4	THO complex 4	9	22	31.1%	26888
[View Peptides]	SNRPA1	small nuclear ribonucleoprotein polypeptide A'	5	7	31.0%	28416
[View Peptides]	SNRPD3	small nuclear ribonucleoprotein D3 polypeptide 18kDa	3	9	31.0%	13916
[View Peptides]	RPLP0	ribosomal protein, large, P0	11	21	30.9%	34274
[View Peptides]	HNRNPU	heterogeneous nuclear ribonucleoprotein U (scaffold attachment factor A)	23	69	30.6%	90480
[View Peptides]	KRT1	keratin 1 (epidermolytic hyperkeratosis)	28	61	30.0%	66018
[View Peptides]	RPL37A	ribosomal protein L37a	2	5	29.3%	10275
[View Peptides]	gi|545257, gi|21...	sp|P35527|K1CI_HUMAN Keratin, type I cytoskeletal 9 (Cytokeratin 9) (K9) (CK 9) , gi|545257|gb|AAC60...	11	26	29.1%	61987
[View Peptides]	HIST1H4L, HIST4H...	histone cluster 2, H4a, histone cluster 1, H4i, histone cluster 1, H4l, histone cluster 1, H4e, hist...	3	5	29.1%	11367
[View Peptides]	RPL36	ribosomal protein L36	5	9	28.6%	12254
[View Peptides]	CIRBP	cold inducible RNA binding protein	8	50	28.5%	18648
[View Peptides]	SND1	staphylococcal nuclease and tudor domain containing 1	24	93	27.7%	101997
[View Peptides]	RPL32	ribosomal protein L32	3	5	27.4%	15860
[View Peptides]	SYNCRIP	synaptotagmin binding, cytoplasmic RNA interacting protein	12	32	27.3%	69633
[View Peptides]	APOBEC3C	apolipoprotein B mRNA editing enzyme, catalytic polypeptide-like 3C	3	3	26.8%	22826
[View Peptides]	C14orf166	chromosome 14 open reading frame 166	7	15	26.6%	28068
[View Peptides]	PPP1CA	protein phosphatase 1, catalytic subunit, alpha isoform	6	42	26.4%	37512
[View Peptides]	DDX5	DEAD (Asp-Glu-Ala-Asp) box polypeptide 5	18	52	26.2%	69148
[View Peptides]	RPS3	ribosomal protein S3	5	9	25.9%	26688

[View Peptides]	HNRNPA1	heterogeneous nuclear ribonucleoprotein A1	9	25	25.8%	38846
[View Peptides]	RPL7A	ribosomal protein L7a	8	21	25.6%	29996
[View Peptides]	G3BP1	GTPase activating protein (SH3 domain) binding protein 1	11	88	25.5%	52164
[View Peptides]	RPL18	ribosomal protein L18	6	23	25.5%	21634
[View Peptides]	IQGAP1	IQ motif containing GTPase activating protein 1	31	71	25.3%	189251
[View Peptides]	gi|41019521	sp|Q9NR30|DD21_HUMAN Nucleolar RNA helicase II (Nucleolar RNA helicase Gu) (RH II/Gu) (DEAD-box prot...	16	38	25.2%	87357
[View Peptides]	CCT8	chaperonin containing TCP1, subunit 8 (theta)	12	17	25.2%	59621
[View Peptides]	TUBA1C	tubulin, alpha 1c	9	24	25.2%	49895
[View Peptides]	NPM1	nucleophosmin (nucleolar phosphoprotein B23, numatrin)	6	42	25.2%	32575
[View Peptides]	TUBA1A	tubulin, alpha 1a	9	24	25.1%	50136
[View Peptides]	EFTUD2	elongation factor Tu GTP binding domain containing 2	16	31	25.1%	109436
[View Peptides]	TUBA4A, TUBA1B	tubulin, alpha 1b, tubulin, alpha 4a	9	24	25.1%	50152
[View Peptides]	YWHAE	tyrosine 3-monooxygenase/tryptophan 5-monooxygenase activation protein, epsilon polypeptide	5	6	25.1%	29174
[View Peptides]	RPL23	ribosomal protein L23	2	6	25.0%	14865
[View Peptides]	FLNA	filamin A, alpha (actin binding protein 280)	44	100	24.9%	280759
[View Peptides]	GRSF1	G-rich RNA sequence binding factor 1	7	11	24.8%	50170
[View Peptides]	RPL12	ribosomal protein L12	4	7	24.8%	17819
[View Peptides]	SEPT11	septin 11	12	39	24.7%	49398
[View Peptides]	DYNLL1	dynein, light chain, LC8-type 1	2	2	24.7%	10366
[View Peptides]	ALDOA	aldolase A, fructose-bisphosphate	6	10	24.5%	39420
[View Peptides]	SERBP1	SERPINE1 mRNA binding protein 1	9	40	24.5%	44965
[View Peptides]	TUBB	tubulin, beta	9	19	24.5%	49671
[View Peptides]	MAP4	microtubule-associated protein 4	21	38	24.5%	121019
[View Peptides]	PSMA7	proteasome (prosome, macropain) subunit, alpha type, 7	4	5	24.2%	27887
[View Peptides]	ACTN4	actinin, alpha 4	16	29	24.1%	104854
[View Peptides]	SEPT10	septin 10	9	26	23.8%	59982
[View Peptides]	YBX1	Y box binding protein 1	4	10	23.8%	35924
[View Peptides]	ENO1	enolase 1, (alpha)	7	12	23.5%	47169
[View Peptides]	YWHAZ	tyrosine 3-monooxygenase/tryptophan 5-monooxygenase activation protein, zeta polypeptide	4	7	23.3%	27745
[View Peptides]	PPP1CC	protein phosphatase 1, catalytic subunit, gamma isoform	5	41	23.2%	36984
[View Peptides]	HIST1H1D	histone cluster 1, H1d	5	10	23.1%	22350
[View Peptides]	TUBA4A	tubulin, alpha 4a	8	16	23.0%	49924
[View Peptides]	FUS	fusion (involved in t(12;16) in malignant liposarcoma)	26	185	22.8%	53426

[View Peptides]	PCBP1	poly(rC) binding protein 1	5	13	22.8%	37498
[View Peptides]	SEPT6	septin 6	10	29	22.6%	49717
[View Peptides]	RFC4	replication factor C (activator 1) 4, 37kDa	6	6	22.6%	39682
[View Peptides]	RPL27A	ribosomal protein L27a	4	5	22.3%	16561
[View Peptides]	CKB	creatine kinase, brain	5	6	22.3%	42644
[View Peptides]	CCT2	chaperonin containing TCP1, subunit 2 (beta)	7	10	22.2%	57488
[View Peptides]	gi|21619210, gi|...	pir||MOHULP myosin regulatory light chain, placental - human , gi|5453740|ref|NP_006462.1| myosin re...	2	3	22.2%	19794
[View Peptides]	PKM2	pyruvate kinase, muscle	7	13	22.0%	57937
[View Peptides]	TUBB2C	tubulin, beta 2C	8	10	21.8%	49831
[View Peptides]	RBM3	RNA binding motif (RNP1, RRM) protein 3	3	5	21.7%	17170
[View Peptides]	XRN2	5'-3' exoribonuclease 2	17	44	21.5%	108583
[View Peptides]	ACTN1	actinin, alpha 1	13	17	21.5%	103058
[View Peptides]	SFRS3	splicing factor, arginine/serine-rich 3	4	15	21.3%	19330
[View Peptides]	RPS4X	ribosomal protein S4, X-linked	4	6	21.3%	29598
[View Peptides]	EXOSC2	exosome component 2	4	4	21.2%	32789
[View Peptides]	PGK1	phosphoglycerate kinase 1	5	9	21.1%	44615
[View Peptides]	UBC, UBB, UBA52	ubiquitin C, ubiquitin B, ubiquitin A-52 residue ribosomal protein fusion product 1	2	5	21.1%	8565
[View Peptides]	CCT7	chaperonin containing TCP1, subunit 7 (eta)	9	16	21.0%	59367
[View Peptides]	KRT2	keratin 2 (epidermal ichthyosis bullosa of Siemens)	15	32	20.8%	65865
[View Peptides]	RPL4	ribosomal protein L4	11	20	20.8%	47697
[View Peptides]	TPI1	triosephosphate isomerase 1	3	9	20.5%	26669
[View Peptides]	HSPA8	heat shock 70kDa protein 8	9	19	20.4%	70898
[View Peptides]	SFRS9	splicing factor, arginine/serine-rich 9	4	20	20.4%	25542
[View Peptides]	SPTAN1	spectrin, alpha, non-erythrocytic 1 (alpha-fodrin)	37	68	20.3%	284526
[View Peptides]	RBM39	RNA binding motif protein 39	9	22	20.2%	59380
[View Peptides]	PPP1CB	protein phosphatase 1, catalytic subunit, beta isoform	4	34	20.2%	37187
[View Peptides]	EIF6	eukaryotic translation initiation factor 6	3	6	20.0%	26599
[View Peptides]	RPL18A	ribosomal protein L18a	4	6	19.9%	20762
[View Peptides]	ACTL6A	actin-like 6A	6	7	19.8%	47461
[View Peptides]	RPL24	ribosomal protein L24	4	8	19.7%	17779
[View Peptides]	HNRNPL	heterogeneous nuclear ribonucleoprotein L	7	17	19.4%	60187
[View Peptides]	S100A8	S100 calcium binding protein A8	2	2	19.4%	10834
[View Peptides]	RPL9	ribosomal protein L9	4	8	19.3%	21863
[View Peptides]	PSMA4	proteasome (prosome, macropain) subunit, alpha type, 4	3	4	19.2%	29484
[View Peptides]	RPS15A	ribosomal protein S15a	3	5	19.2%	14839
[View Peptides]	RPS26	ribosomal protein S26	2	4	19.1%	13015

[View Peptides]	HSPA5	heat shock 70kDa protein 5 (glucose-regulated protein, 78kDa)	9	11	19.1%	72333
[View Peptides]	VCP	valosin-containing protein	10	12	19.1%	89322
[View Peptides]	CORO1C	coronin, actin binding protein, 1C	9	20	19.0%	53249
[View Peptides]	NOLA2	nucleolar protein family A, member 2 (H/ACA small nucleolar RNPs)	2	4	19.0%	17201
[View Peptides]	U2AF2	U2 small nuclear RNA auxiliary factor 2	6	17	18.9%	53501
[View Peptides]	DDX17	DEAD (Asp-Glu-Ala-Asp) box polypeptide 17	12	30	18.8%	72372
[View Peptides]	POLR1C	polymerase (RNA) I polypeptide C, 30kDa	4	6	18.8%	39250
[View Peptides]	CFL1	cofilin 1 (non-muscle)	2	3	18.7%	18502
[View Peptides]	CFL2	cofilin 2 (muscle)	2	2	18.7%	18737
[View Peptides]	CDC5L	CDC5 cell division cycle 5-like (S. pombe)	11	17	18.7%	92251
[View Peptides]	SPTBN1	spectrin, beta, non-erythrocytic 1	31	51	18.6%	274630
[View Peptides]	MYL6	myosin, light chain 6, alkali, smooth muscle and non-muscle	3	3	18.5%	16930
[View Peptides]	GNB2L1	guanine nucleotide binding protein (G protein), beta polypeptide 2-like 1	4	4	18.3%	35077
[View Peptides]	LRPPRC	leucine-rich PPR-motif containing	15	23	18.2%	145201
[View Peptides]	EXOSC3	exosome component 3	3	4	18.2%	29572
[View Peptides]	CSNK2B	casein kinase 2, beta polypeptide	2	3	18.1%	24942
[View Peptides]	RFC2	replication factor C (activator 1) 2, 40kDa	4	7	18.1%	39157
[View Peptides]	EWSR1	Ewing sarcoma breakpoint region 1	13	52	18.0%	68478
[View Peptides]	ACTC1	actin, alpha, cardiac muscle 1	10	34	18.0%	42019
[View Peptides]	ACTA1	actin, alpha 1, skeletal muscle	10	34	18.0%	42051
[View Peptides]	ACTA2	actin, alpha 2, smooth muscle, aorta	10	34	18.0%	42009
[View Peptides]	PCNA	proliferating cell nuclear antigen	3	5	18.0%	28769
[View Peptides]	EIF2S2	eukaryotic translation initiation factor 2, subunit 2 beta, 38kDa	4	5	17.7%	38388
[View Peptides]	EMG1	EMG1 nucleolar protein homolog (S. cerevisiae)	3	5	17.6%	26720
[View Peptides]	PPIH	peptidylprolyl isomerase H (cyclophilin H)	2	2	17.5%	19208
[View Peptides]	THOC1	THO complex 1	8	13	17.5%	75666
[View Peptides]	NOL1	nucleolar protein 1, 120kDa	11	19	17.5%	94078
[View Peptides]	PSMA5	proteasome (prosome, macropain) subunit, alpha type, 5	3	3	17.4%	26411
[View Peptides]	SFRS5	splicing factor, arginine/serine-rich 5	3	5	17.3%	31264
[View Peptides]	MORC2	MORC family CW-type zinc finger 2	11	17	17.2%	110724
[View Peptides]	RPS5	ribosomal protein S5	2	3	17.2%	22876
[View Peptides]	PRDX6	peroxiredoxin 6	2	2	17.0%	25035
[View Peptides]	RPL21	ribosomal protein L21	3	6	16.9%	18565
[View Peptides]	RPL3	ribosomal protein L3	9	14	16.9%	46109
[View Peptides]	TMPO	thymopoietin	6	6	16.9%	75492

[View Peptides]	CKAP5	cytoskeleton associated protein 5	34	46	16.9%	225507
[View Peptides]	TOP2A	topoisomerase (DNA) II alpha 170kDa	18	33	16.8%	174384
[View Peptides]	SAFB2	scaffold attachment factor B2	14	22	16.5%	107474
[View Peptides]	RPS2	ribosomal protein S2	4	4	16.4%	31324
[View Peptides]	WDR74	WD repeat domain 74	4	4	16.1%	42441
[View Peptides]	TCP1	t-complex 1	6	6	16.0%	60344
[View Peptides]	TARDBP	TAR DNA binding protein	6	7	15.9%	44740
[View Peptides]	EXOSC10	exosome component 10	10	15	15.9%	100831
[View Peptides]	CD2BP2	CD2 (cytoplasmic tail) binding protein 2	3	5	15.8%	37646
[View Peptides]	SNRPG	small nuclear ribonucleoprotein polypeptide G	2	2	15.8%	8496
[View Peptides]	DDX23	DEAD (Asp-Glu-Ala-Asp) box polypeptide 23	10	12	15.7%	95647
[View Peptides]	RPS6	ribosomal protein S6	3	5	15.7%	28681
[View Peptides]	ASCC3L1	activating signal cointegrator 1 complex subunit 3-like 1	23	36	15.6%	244505
[View Peptides]	RPL14	ribosomal protein L14	5	8	15.5%	23290
[View Peptides]	TAF15	TAF15 RNA polymerase II, TATA box binding protein (TBP)-associated factor, 68kDa	7	55	15.5%	61830
[View Peptides]	KRT6C	keratin 6C	8	14	15.4%	60025
[View Peptides]	PRPF19	PRP19/PSO4 pre-mRNA processing factor 19 homolog (S. cerevisiae)	6	16	15.3%	55181
[View Peptides]	TERF2IP	telomeric repeat binding factor 2, interacting protein	4	4	15.3%	44260
[View Peptides]	RPL7	ribosomal protein L7	5	8	15.3%	29226
[View Peptides]	CNBP	CCHC-type zinc finger, nucleic acid binding protein	3	36	15.3%	19463
[View Peptides]	KRT16	keratin 16 (focal non-epidermolytic palmoplantar keratoderma)	7	12	15.2%	51268
[View Peptides]	RPS15	ribosomal protein S15	2	2	15.2%	17040
[View Peptides]	RUVBL2	RuvB-like 2 (E. coli)	5	8	15.1%	51157
[View Peptides]	DHX9	DEAH (Asp-Glu-Ala-His) box polypeptide 9	15	41	15.1%	140881
[View Peptides]	SAFB	scaffold attachment factor B	11	18	15.0%	102640
[View Peptides]	ELAVL1	ELAV (embryonic lethal, abnormal vision, Drosophila)-like 1 (Hu antigen R)	4	7	15.0%	36092
[View Peptides]	EXOSC5	exosome component 5	2	3	14.9%	25249
[View Peptides]	POLR2E	polymerase (RNA) II (DNA directed) polypeptide E, 25kDa	2	4	14.8%	24611
[View Peptides]	gi|87654, gi|929...	gi|929663|emb|CAA27544.1| unnamed protein product [Homo sapiens], pir||A24016 heterogeneous ribonucl...	2	8	14.7%	24198
[View Peptides]	RPL13	ribosomal protein L13	4	6	14.7%	24261
[View Peptides]	BCAS2	breast carcinoma amplified sequence 2	2	4	14.7%	26131
[View Peptides]	PA2G4	proliferation-associated 2G4, 38kDa	4	8	14.7%	43787
[View Peptides]	TUBB3, TUBB4	tubulin, beta 4, tubulin, beta 3	6	10	14.7%	50433
[View Peptides]	CCT4	chaperonin containing TCP1, subunit 4 (delta)	5	9	14.7%	57924

[View Peptides]	gi|107222, gi|48...	ref|NP_005372.1| nucleolin [Homo sapiens] , gi|189306|gb|AAA59954.1| nucleolin, gi|128841|sp|P19338|...	8	18	14.7%	76344
[View Peptides]	TOP1	topoisomerase (DNA) I	11	16	14.6%	90726
[View Peptides]	RPL34	ribosomal protein L34	2	2	14.5%	13293
[View Peptides]	RPL35A	ribosomal protein L35a	2	4	14.5%	12538
[View Peptides]	PSMB5	proteasome (prosome, macropain) subunit, beta type, 5	2	2	14.4%	22897
[View Peptides]	DNAJC8	DnaJ (Hsp40) homolog, subfamily C, member 8	2	3	14.2%	29842
[View Peptides]	KPNA2	karyopherin alpha 2 (RAG cohort 1, importin alpha 1)	4	5	14.2%	57862
[View Peptides]	TPM3	tropomyosin 3	5	9	14.1%	32819
[View Peptides]	RPL8	ribosomal protein L8	4	5	14.0%	28025
[View Peptides]	ANXA2	annexin A2	3	4	13.9%	38604
[View Peptides]	WDR3	WD repeat domain 3	7	11	13.8%	106099
[View Peptides]	RPP30	ribonuclease P/MRP 30kDa subunit	2	2	13.8%	29321
[View Peptides]	HDGF	hepatoma-derived growth factor (high-mobility group protein 1-like)	2	3	13.8%	26788
[View Peptides]	RPS19	ribosomal protein S19	2	2	13.8%	16060
[View Peptides]	RPL15	ribosomal protein L15	3	8	13.7%	24146
[View Peptides]	HNRPK	heterogeneous nuclear ribonucleoprotein K	5	6	13.6%	50976
[View Peptides]	RBBP4	retinoblastoma binding protein 4	5	7	13.6%	47656
[View Peptides]	YBX2	Y box binding protein 2	3	7	13.5%	38560
[View Peptides]	HIST1H2BL	histone cluster 1, H2bl	2	3	13.5%	13952
[View Peptides]	HIST2H2BE	histone cluster 2, H2be	2	3	13.5%	13920
[View Peptides]	gi|119623521, gi...	gi|7387741|sp|Q99877|H2B1N_HUMAN Histone H2B type 1-N (H2B.d) (H2B/d), gi|72533606|gb|AAI01412.1| Hi...	2	3	13.5%	13922
[View Peptides]	HIST3H2BB	histone cluster 3, H2bb	2	3	13.5%	13908
[View Peptides]	HIST1H2BJ	histone cluster 1, H2bj	2	3	13.5%	13904
[View Peptides]	HIST1H2BD	histone cluster 1, H2bd	2	3	13.5%	13936
[View Peptides]	HIST1H2BK	histone cluster 1, H2bk	2	3	13.5%	13890
[View Peptides]	H2BFS	H2B histone family, member S	2	3	13.5%	13944
[View Peptides]	HIST1H2BO, HIST1H2BE	histone cluster 1, H2bo, histone cluster 1, H2be	2	3	13.5%	13906
[View Peptides]	HIST1H2BH	histone cluster 1, H2bh	2	3	13.5%	13892
[View Peptides]	WBSCR22	Williams Beuren syndrome chromosome region 22	2	2	13.5%	31880
[View Peptides]	gi|22770649, gi|...	emb|CAC04135.1| dJ221C16.8 (novel histone 2B family member) [Homo sapiens] , gi|9863666|emb|CAC04130...	2	3	13.5%	13906
[View Peptides]	PSMB4	proteasome (prosome, macropain) subunit, beta type, 4	2	5	13.3%	29192
[View Peptides]	RPS23	ribosomal protein S23	3	3	13.3%	15808
[View Peptides]	PDIA6	protein disulfide isomerase family A, member 6	3	4	13.2%	48121

[View Peptides]	TCOF1	Treacher Collins-Franceschetti syndrome 1	13	22	13.0%	144312
[View Peptides]	EXOSC9	exosome component 9	4	5	13.0%	46978
[View Peptides]	HNRPA3	heterogeneous nuclear ribonucleoprotein A3	4	10	13.0%	39595
[View Peptides]	CCT3	chaperonin containing TCP1, subunit 3 (gamma)	5	6	12.7%	60534
[View Peptides]	GPR137, CAPRIN1	cell cycle associated protein 1, G protein-coupled receptor 137	9	28	12.6%	72752
[View Peptides]	MTA2	metastasis associated 1 family, member 2	5	5	12.6%	75023
[View Peptides]	RPL27	ribosomal protein L27	3	5	12.5%	15798
[View Peptides]	SNRPB	small nuclear ribonucleoprotein polypeptides B and B1	4	11	12.5%	24610
[View Peptides]	SNRPN, SNURF	SNRPN upstream reading frame, small nuclear ribonucleoprotein polypeptide N	4	11	12.5%	24614
[View Peptides]	TPM4	tropomyosin 4	4	10	12.5%	28522
[View Peptides]	RPS3A	ribosomal protein S3A	4	7	12.5%	29945
[View Peptides]	LSM14A	LSM14A, SCD6 homolog A (S. cerevisiae)	5	13	12.5%	50530
[View Peptides]	CTTN	cortactin	3	4	12.5%	61636
[View Peptides]	RPL11	ribosomal protein L11	2	2	12.4%	20252
[View Peptides]	RPS9	ribosomal protein S9	3	4	12.4%	22591
[View Peptides]	EEF1B2	eukaryotic translation elongation factor 1 beta 2	2	2	12.4%	24764
[View Peptides]	AURKA	aurora kinase A	3	4	12.4%	45809
[View Peptides]	CMAS	cytidine monophosphate N-acetylneuraminic acid synthetase	4	5	12.4%	48379
[View Peptides]	FLNB	filamin B, beta (actin binding protein 278)	19	27	12.4%	278193
[View Peptides]	MED4	mediator complex subunit 4	2	2	12.2%	29745
[View Peptides]	PSMA1	proteasome (prosome, macropain) subunit, alpha type, 1	3	3	12.2%	29556
[View Peptides]	PRDX1	peroxiredoxin 1	2	2	12.1%	22110
[View Peptides]	HNRNPC	heterogeneous nuclear ribonucleoprotein C (C1/C2)	4	5	12.1%	33688
[View Peptides]	KRT14	keratin 14 (epidermolysis bullosa simplex, Dowling-Meara, Koebner)	6	9	12.1%	51622
[View Peptides]	SF3A3	splicing factor 3a, subunit 3, 60kDa	5	9	12.0%	58849
[View Peptides]	BAT1	HLA-B associated transcript 1	3	10	11.9%	48991
[View Peptides]	TRIM28	tripartite motif-containing 28	7	15	11.9%	88550
[View Peptides]	KIF23	kinesin family member 23	6	8	11.9%	98105
[View Peptides]	SF3B3	splicing factor 3b, subunit 3, 130kDa	11	21	11.9%	135592
[View Peptides]	G3BP2	GTPase activating protein (SH3 domain) binding protein 2	4	16	11.8%	54111
[View Peptides]	IGHG1	immunoglobulin heavy constant gamma 1 (G1m marker)	2	2	11.8%	36106
[View Peptides]	ACAT1	acetyl-Coenzyme A acetyltransferase 1 (acetoacetyl Coenzyme A thiolase)	3	4	11.7%	45200
[View Peptides]	DDB2	damage-specific DNA binding protein 2, 48kDa	3	5	11.7%	47864

[View Peptides]	EIF2S3	eukaryotic translation initiation factor 2, subunit 3 gamma, 52kDa	4	4	11.7%	51110
[View Peptides]	CCT6A	chaperonin containing TCP1, subunit 6A (zeta 1)	4	7	11.7%	58024
[View Peptides]	BUB3	BUB3 budding uninhibited by benzimidazoles 3 homolog (yeast)	3	3	11.6%	37155
[View Peptides]	ACTR3	ARP3 actin-related protein 3 homolog (yeast)	3	3	11.5%	47371
[View Peptides]	PAK1IP1	PAK1 interacting protein 1	3	5	11.5%	43964
[View Peptides]	PCBP2	poly(rC) binding protein 2	3	8	11.5%	38580
[View Peptides]	DRG1	developmentally regulated GTP binding protein 1	3	3	11.4%	40542
[View Peptides]	ALDOC	aldolase C, fructose-bisphosphate	3	11	11.3%	39456
[View Peptides]	FEN1	flap structure-specific endonuclease 1	3	5	11.3%	42593
[View Peptides]	PLRG1	pleiotropic regulator 1 (PRL1 homolog, Arabidopsis)	4	7	11.3%	57194
[View Peptides]	HSPD1	heat shock 60kDa protein 1 (chaperonin)	3	4	11.3%	61055
[View Peptides]	PDIA4	protein disulfide isomerase family A, member 4	4	4	11.3%	72933
[View Peptides]	RPL6	ribosomal protein L6	3	3	11.1%	32728
[View Peptides]	EBNA1BP2	EBNA1 binding protein 2	2	6	11.1%	34820
[View Peptides]	BCLAF1	BCL2-associated transcription factor 1	7	13	11.1%	106122
[View Peptides]	RBMX	RNA binding motif protein, X-linked	6	24	11.0%	42332
[View Peptides]	YWHAH	tyrosine 3-monooxygenase/tryptophan 5-monooxygenase activation protein, eta polypeptide	2	3	11.0%	28219
[View Peptides]	YWHAB	tyrosine 3-monooxygenase/tryptophan 5-monooxygenase activation protein, beta polypeptide	2	3	11.0%	28082
[View Peptides]	YWHAQ	tyrosine 3-monooxygenase/tryptophan 5-monooxygenase activation protein, theta polypeptide	2	3	11.0%	27764
[View Peptides]	RPL26L1	ribosomal protein L26-like 1	2	4	11.0%	17256
[View Peptides]	RPL26	ribosomal protein L26	2	4	11.0%	17258
[View Peptides]	GTF3C5	general transcription factor IIIC, polypeptide 5, 63kDa	4	4	11.0%	59571
[View Peptides]	YWHAG	tyrosine 3-monooxygenase/tryptophan 5-monooxygenase activation protein, gamma polypeptide	2	3	10.9%	28303
[View Peptides]	gi|59797947, gi|...	gi|59797947|sp|Q8IUH3|DRBP1_HUMAN Developmentally-regulated RNA-binding protein 1 (RB-1), gi|2283132...	3	5	10.9%	53503
[View Peptides]	HDAC2	histone deacetylase 2	3	4	10.9%	55364
[View Peptides]	SF3A1	splicing factor 3a, subunit 1, 120kDa	7	16	10.8%	88886
[View Peptides]	TMOD3	tropomodulin 3 (ubiquitous)	2	2	10.8%	39595
[View Peptides]	PRPF8	PRP8 pre-mRNA processing factor 8 homolog (S. cerevisiae)	18	26	10.6%	273599
[View Peptides]	DNTTIP1	deoxynucleotidyltransferase, terminal, interacting protein 1	2	2	10.6%	37013
[View Peptides]	PABPN1	poly(A) binding protein, nuclear 1	3	6	10.5%	32749
[View Peptides]	POLR1A	polymerase (RNA) I polypeptide A, 194kDa	11	14	10.4%	194190
[View Peptides]	SF3B1	splicing factor 3b, subunit 1, 155kDa	9	14	10.4%	145815

[View Peptides]	GTF2H3	general transcription factor IIH, polypeptide 3, 34kDa	2	2	10.4%	34378
[View Peptides]	H1FX	H1 histone family, member X	2	4	10.3%	22487
[View Peptides]	IFI16	interferon, gamma-inducible protein 16	6	8	10.3%	88275
[View Peptides]	SF3B2	splicing factor 3b, subunit 2, 145kDa	5	9	10.2%	97657
[View Peptides]	ERCC3	excision repair cross-complementing rodent repair deficiency, complementation group 3 (xeroderma pig...	5	7	10.2%	89278
[View Peptides]	SFRS12	splicing factor, arginine/serine-rich 12	3	3	10.2%	59380
[View Peptides]	RPSA	ribosomal protein SA	2	2	10.2%	32854
[View Peptides]	NOLA1	nucleolar protein family A, member 1 (H/ACA small nucleolar RNPs)	2	2	10.1%	22348
[View Peptides]	SNRP70	small nuclear ribonucleoprotein 70kDa polypeptide (RNP antigen)	4	8	10.1%	51557
[View Peptides]	SHMT2	serine hydroxymethyltransferase 2 (mitochondrial)	3	5	10.1%	55993
[View Peptides]	CIRH1A	cirrhosis, autosomal recessive 1A (cirhin)	4	7	10.1%	76890
[View Peptides]	DCD	dermcidin	2	3	10.0%	11284
[View Peptides]	EXOSC7	exosome component 7	2	3	10.0%	31835
[View Peptides]	FBL	fibrillarin	2	5	10.0%	33784
[View Peptides]	CCT5	chaperonin containing TCP1, subunit 5 (epsilon)	4	5	10.0%	59671
[View Peptides]	MYH9	myosin, heavy chain 9, non-muscle	11	21	9.9%	226530
[View Peptides]	SNW1	SNW domain containing 1	3	3	9.9%	61495
[View Peptides]	gi|2506774	gi|2506774|sp|P05787|K2C8_HUMAN Keratin, type II cytoskeletal 8 (Cytokeratin-8) (CK-8) (Keraton-8) (...	4	6	9.9%	53674
[View Peptides]	RRS1	RRS1 ribosome biogenesis regulator homolog (S. cerevisiae)	2	3	9.9%	41193
[View Peptides]	C20orf77	chromosome 20 open reading frame 77	2	2	9.8%	36900
[View Peptides]	SEPT5	septin 5	2	7	9.8%	42777
[View Peptides]	VRK1	vaccinia related kinase 1	3	3	9.8%	45476
[View Peptides]	TOP2B	topoisomerase (DNA) II beta 180kDa	11	19	9.8%	183266
[View Peptides]	SFRS7	splicing factor, arginine/serine-rich 7, 35kDa	2	10	9.7%	27367
[View Peptides]	DPF2	D4, zinc and double PHD fingers family 2	2	2	9.7%	44156
[View Peptides]	COIL	coilin	4	4	9.5%	62608
[View Peptides]	PABPC1	poly(A) binding protein, cytoplasmic 1	5	6	9.4%	70671
[View Peptides]	RPL13A	ribosomal protein L13a	2	4	9.4%	23577
[View Peptides]	SMARCB1	SWI/SNF related, matrix associated, actin dependent regulator of chromatin, subfamily b, member 1	2	3	9.4%	44141
[View Peptides]	HIST1H1B	histone cluster 1, H1b	2	4	9.3%	22580
[View Peptides]	LDHB	lactate dehydrogenase B	2	4	9.3%	36639
[View Peptides]	HNRPD	heterogeneous nuclear ribonucleoprotein D (AU-rich element RNA binding protein 1, 37kDa)	2	8	9.3%	38434
[View Peptides]	ABCF1	ATP-binding cassette, sub-family F (GCN20), member 1	6	6	9.3%	95926
[View Peptides]	DDB1	damage-specific DNA binding protein 1, 127kDa	8	11	9.2%	126968

[View Peptides]	VIL1	villin 1	6	6	9.2%	92695
[View Peptides]	RPL10A	ribosomal protein L10a	2	2	9.2%	24831
[View Peptides]	WDR1	WD repeat domain 1	3	7	8.9%	66194
[View Peptides]	DHX15	DEAH (Asp-Glu-Ala-His) box polypeptide 15	5	12	8.9%	90933
[View Peptides]	WDR36	WD repeat domain 36	6	6	8.9%	105322
[View Peptides]	gi|550013, gi|10...	gi|550013|gb|AAA85654.1| ribosomal protein L5, pir||S55912 ribosomal protein L5, cytosolic - human ,...	2	4	8.8%	34448
[View Peptides]	WDR5	WD repeat domain 5	2	2	8.7%	36589
[View Peptides]	LMNA	lamin A/C	4	9	8.7%	74140
[View Peptides]	KRT5	keratin 5 (epidermolysis bullosa simplex, Dowling-Meara/Kobner/Weber-Cockayne types)	6	12	8.6%	62447
[View Peptides]	POLDIP3	polymerase (DNA-directed), delta interacting protein 3	2	2	8.6%	46089
[View Peptides]	TGM3	transglutaminase 3 (E polypeptide, protein-glutamine-gamma-glutamyltransferase)	3	7	8.5%	76632
[View Peptides]	CSTF2	cleavage stimulation factor, 3' pre-RNA, subunit 2, 64kDa	3	4	8.5%	60959
[View Peptides]	BCKDK	branched chain ketoacid dehydrogenase kinase	3	3	8.5%	46360
[View Peptides]	RFC5	replication factor C (activator 1) 5, 36.5kDa	2	3	8.5%	38497
[View Peptides]	EIF4A1	eukaryotic translation initiation factor 4A, isoform 1	2	2	8.4%	46154
[View Peptides]	GTPBP4	GTP binding protein 4	4	4	8.4%	73965
[View Peptides]	NOLC1	nucleolar and coiled-body phosphoprotein 1	5	9	8.3%	73720
[View Peptides]	RPL23A	ribosomal protein L23a	2	2	8.3%	17695
[View Peptides]	PWP2	PWP2 periodic tryptophan protein homolog (yeast)	5	8	8.1%	102451
[View Peptides]	KIF2C	kinesin family member 2C	4	4	8.1%	81313
[View Peptides]	PABPC4	poly(A) binding protein, cytoplasmic 4 (inducible form)	4	5	8.1%	70783
[View Peptides]	NXF1	nuclear RNA export factor 1	3	3	8.1%	70182
[View Peptides]	PDIA3	protein disulfide isomerase family A, member 3	3	3	8.1%	56782
[View Peptides]	PSMD12	proteasome (prosome, macropain) 26S subunit, non-ATPase, 12	2	2	8.1%	52904
[View Peptides]	EEF1G	eukaryotic translation elongation factor 1 gamma	2	3	8.0%	50119
[View Peptides]	USP10	ubiquitin specific peptidase 10	4	4	8.0%	87134
[View Peptides]	CSTF2T	cleavage stimulation factor, 3' pre-RNA, subunit 2, 64kDa, tau variant	3	4	8.0%	64436
[View Peptides]	MYBBP1A	MYB binding protein (P160) 1a	6	9	7.9%	148854
[View Peptides]	PRPF40A	PRP40 pre-mRNA processing factor 40 homolog A (S. cerevisiae)	5	9	7.9%	108805
[View Peptides]	CPSF2	cleavage and polyadenylation specific factor 2, 100kDa	3	3	7.9%	88487
[View Peptides]	HNRPM	heterogeneous nuclear ribonucleoprotein M	4	6	7.9%	77516
[View Peptides]	CDSN	corneodesmosin	2	4	7.9%	51495
[View Peptides]	DAZAP1	DAZ associated protein 1	2	2	7.9%	43383

[View Peptides]	RBBP5	retinoblastoma binding protein 5	2	2	7.8%	59082
[View Peptides]	FSCN1	fascin homolog 1, actin-bundling protein (Strongylocentrotus purpuratus)	2	2	7.7%	54530
[View Peptides]	PLK1	polo-like kinase 1 (Drosophila)	3	3	7.6%	68255
[View Peptides]	MSN	moesin	3	3	7.6%	67820
[View Peptides]	WDR57	WD repeat domain 57 (U5 snRNP specific)	2	3	7.6%	39311
[View Peptides]	HSP90AB1	heat shock protein 90kDa alpha (cytosolic), class B member 1	4	6	7.5%	83264
[View Peptides]	KHDRBS1	KH domain containing, RNA binding, signal transduction associated 1	4	6	7.4%	48227
[View Peptides]	MTA1	metastasis associated 1	3	5	7.4%	80788
[View Peptides]	UBAP2L	ubiquitin associated protein 2-like	4	6	7.4%	103930
[View Peptides]	IFIT5	interferon-induced protein with tetratricopeptide repeats 5	2	2	7.3%	55847
[View Peptides]	METAP2	methionyl aminopeptidase 2	2	3	7.3%	52892
[View Peptides]	NAT10	N-acetyltransferase 10	4	4	7.2%	115704
[View Peptides]	LDHA	lactate dehydrogenase A	2	2	7.2%	36689
[View Peptides]	MBD2	methyl-CpG binding domain protein 2	2	2	7.1%	43255
[View Peptides]	CHD4	chromodomain helicase DNA binding protein 4	9	11	7.1%	217989
[View Peptides]	HSPA1A, HSPA1B	heat shock 70kDa protein 1B, heat shock 70kDa protein 1A	3	5	6.9%	70052
[View Peptides]	ATIC	5-aminoimidazole-4-carboxamide ribonucleotide formyltransferase/IMP cyclohydrolase	2	3	6.9%	64616
[View Peptides]	VIM	vimentin	2	2	6.9%	53652
[View Peptides]	DDX39	DEAD (Asp-Glu-Ala-Asp) box polypeptide 39	2	4	6.8%	49130
[View Peptides]	IK	IK cytokine, down-regulator of HLA II	2	2	6.8%	65630
[View Peptides]	GMIP	GEM interacting protein	4	5	6.8%	106733
[View Peptides]	KRT17	keratin 17	4	6	6.7%	48106
[View Peptides]	SMARCD1	SWI/SNF related, matrix associated, actin dependent regulator of chromatin, subfamily d, member 1	2	3	6.7%	54945
[View Peptides]	ROD1	ROD1 regulator of differentiation 1 (S. pombe)	3	5	6.7%	56502
[View Peptides]	PPP2R1B	protein phosphatase 2 (formerly 2A), regulatory subunit A, beta isoform	2	2	6.7%	66202
[View Peptides]	RAD21	RAD21 homolog (S. pombe)	2	2	6.7%	71690
[View Peptides]	HSP90B1	heat shock protein 90kDa beta (Grp94), member 1	3	4	6.7%	92469
[View Peptides]	TBL1XR1	transducin (beta)-like 1X-linked receptor 1	2	3	6.6%	55595
[View Peptides]	PRMT3	protein arginine methyltransferase 3	2	2	6.6%	59903
[View Peptides]	DDX18	DEAD (Asp-Glu-Ala-Asp) box polypeptide 18	3	3	6.6%	75407
[View Peptides]	ZBTB1	zinc finger and BTB domain containing 1	3	4	6.6%	82016
[View Peptides]	gi|40254816, gi|...	gi|40254816|ref|NP_005339.2| heat shock protein 90kDa alpha (cytosolic), class A member 1 isoform 2 ...	4	4	6.6%	84674
[View Peptides]	DNMT1	DNA (cytosine-5-)-methyltransferase 1	6	7	6.6%	183164

[View Peptides]	NMT1	N-myristoyltransferase 1	2	3	6.5%	56806
[View Peptides]	NOL11	nucleolar protein 11	3	3	6.5%	81124
[View Peptides]	EEF2	eukaryotic translation elongation factor 2	4	5	6.5%	95338
[View Peptides]	BLM	Bloom syndrome	5	5	6.5%	159000
[View Peptides]	MKI67	antigen identified by monoclonal antibody Ki-67	18	22	6.5%	358747
[View Peptides]	MYH10	myosin, heavy chain 10, non-muscle	7	10	6.4%	228937
[View Peptides]	ERCC2	excision repair cross-complementing rodent repair deficiency, complementation group 2 (xeroderma pig...	3	4	6.4%	86909
[View Peptides]	SEPT8	septin 8	3	4	6.4%	55756
[View Peptides]	DDX52	DEAD (Asp-Glu-Ala-Asp) box polypeptide 52	2	2	6.3%	67466
[View Peptides]	RSL1D1	ribosomal L1 domain containing 1	2	3	6.3%	54973
[View Peptides]	HDAC1	histone deacetylase 1	2	3	6.2%	55103
[View Peptides]	SFRS6	splicing factor, arginine/serine-rich 6	2	2	6.1%	39587
[View Peptides]	DEK	DEK oncogene (DNA binding)	2	2	6.1%	42674
[View Peptides]	TOPBP1	topoisomerase (DNA) II binding protein 1	6	10	6.0%	170677
[View Peptides]	SKIV2L2	superkiller viralicidic activity 2-like 2 (S. cerevisiae)	4	4	6.0%	117805
[View Peptides]	gi|119613009, gi...	gi|6562436|emb|CAB62539.1| gamma tubulin ring complex protein [Homo sapiens], gi|22095730|sp|Q9UGJ1|...	2	2	6.0%	76089
[View Peptides]	RBM14	RNA binding motif protein 14	3	5	6.0%	69492
[View Peptides]	CHAF1B	chromatin assembly factor 1, subunit B (p60)	2	2	5.9%	61493
[View Peptides]	TKT	transketolase (Wernicke-Korsakoff syndrome)	2	2	5.9%	67878
[View Peptides]	FTSJ3	FtsJ homolog 3 (E. coli)	3	3	5.9%	96576
[View Peptides]	SYMPK	symplekin	4	8	5.9%	141148
[View Peptides]	HEATR1	HEAT repeat containing 1	8	11	5.9%	242368
[View Peptides]	AHNAK	AHNAK nucleoprotein	10	10	5.8%	312493
[View Peptides]	CHAF1A	chromatin assembly factor 1, subunit A (p150)	4	4	5.8%	105223
[View Peptides]	CALD1	caldesmon 1	3	5	5.8%	93250
[View Peptides]	PPIG	peptidylprolyl isomerase G (cyclophilin G)	3	6	5.8%	88618
[View Peptides]	UTP18	UTP18, small subunit (SSU) processome component, homolog (yeast)	2	2	5.8%	62004
[View Peptides]	DDX3Y	DEAD (Asp-Glu-Ala-Asp) box polypeptide 3, Y-linked	3	6	5.8%	73154
[View Peptides]	DDX3X	DEAD (Asp-Glu-Ala-Asp) box polypeptide 3, X-linked	3	6	5.7%	73244
[View Peptides]	ILF3	interleukin enhancer binding factor 3, 90kDa	3	3	5.7%	95339
[View Peptides]	PPP1R10	protein phosphatase 1, regulatory (inhibitor) subunit 10	4	5	5.7%	99058
[View Peptides]	CHD8	chromodomain helicase DNA binding protein 8	6	12	5.6%	230356
[View Peptides]	RFC1	replication factor C (activator 1) 1, 145kDa	4	7	5.6%	128254
[View Peptides]	PRPF6	PRP6 pre-mRNA processing factor 6 homolog (S. cerevisiae)	3	7	5.6%	106925

[View Peptides]	XRCC6	X-ray repair complementing defective repair in Chinese hamster cells 6 (Ku autoantigen, 70kDa)	2	4	5.6%	69843
[View Peptides]	TERF2	telomeric repeat binding factor 2	2	2	5.6%	55551
[View Peptides]	CUL4A	cullin 4A	3	4	5.5%	76821
[View Peptides]	ACSL4	acyl-CoA synthetase long-chain family member 4	2	2	5.5%	79188
[View Peptides]	THRAP3	thyroid hormone receptor associated protein 3	7	16	5.5%	108694
[View Peptides]	SMARCC2	SWI/SNF related, matrix associated, actin dependent regulator of chromatin, subfamily c, member 2	4	5	5.5%	132879
[View Peptides]	SMARCA4	SWI/SNF related, matrix associated, actin dependent regulator of chromatin, subfamily a, member 4	6	7	5.4%	184585
[View Peptides]	COPB1	coatomer protein complex, subunit beta 1	3	4	5.4%	107139
[View Peptides]	DBN1	drebrin 1	2	4	5.4%	71425
[View Peptides]	POLR1E	polymerase (RNA) I polypeptide E, 53kDa	2	2	5.4%	53962
[View Peptides]	TUBG1	tubulin, gamma 1	2	4	5.3%	51170
[View Peptides]	TUBG2	tubulin, gamma 2	2	4	5.3%	51092
[View Peptides]	TUBGCP2	tubulin, gamma complex associated protein 2	3	4	5.3%	102534
[View Peptides]	MDC1	mediator of DNA damage checkpoint 1	5	6	5.2%	226664
[View Peptides]	CLTC	clathrin, heavy chain (Hc)	5	7	5.1%	191613
[View Peptides]	SRP68	signal recognition particle 68kDa	2	2	5.1%	70730
[View Peptides]	FUBP1	far upstream element (FUSE) binding protein 1	2	2	5.1%	67473
[View Peptides]	DKC1	dyskeratosis congenita 1, dyskerin	2	2	5.1%	57674
[View Peptides]	DSG1	desmoglein 1	3	4	5.0%	113716
[View Peptides]	HMMR	hyaluronan-mediated motility receptor (RHAMM)	2	2	5.0%	84031
[View Peptides]	gi|4758644, gi|3...	gi|4758644|ref|NP_004511.1| kinesin heavy chain member 2 [Homo sapiens], gi|3024057|sp|O00139|KIF2_H...	2	2	4.9%	76927
[View Peptides]	KIF14	kinesin family member 14	4	5	4.9%	186490
[View Peptides]	DIS3	DIS3 mitotic control homolog (S. cerevisiae)	3	3	4.8%	109003
[View Peptides]	LIG3	ligase III, DNA, ATP-dependent	3	4	4.8%	102691
[View Peptides]	GAS2L3	growth arrest-specific 2 like 3	2	2	4.8%	75214
[View Peptides]	LARP1	La ribonucleoprotein domain family, member 1	4	8	4.7%	123510
[View Peptides]	API5	apoptosis inhibitor 5	2	2	4.7%	57561
[View Peptides]	gi|119717, gi|48...	pir||A34400 ezrin [validated] - human , gi|31283|emb|CAA35893.1| unnamed protein product [Homo sapie...	2	2	4.6%	69399
[View Peptides]	SMC3	structural maintenance of chromosomes 3	3	3	4.6%	141541
[View Peptides]	FASN	fatty acid synthase	7	9	4.6%	273397
[View Peptides]	CAST	calpastatin	2	2	4.5%	76501
[View Peptides]	XRCC5	X-ray repair complementing defective repair in Chinese hamster cells 5 (double-strand-break rejoinin...	2	6	4.5%	82705
[View Peptides]	GTF3C4	general transcription factor IIIC, polypeptide 4, 90kDa	2	2	4.5%	92001

[View Peptides]	MKL1, RBM15	RNA binding motif protein 15, megakaryoblastic leukemia (translocation) 1	3	4	4.5%	107188
[View Peptides]	DSP	desmoplakin	8	10	4.5%	331776
[View Peptides]	TUBGCP3	tubulin, gamma complex associated protein 3	2	3	4.4%	103571
[View Peptides]	gi|8923487, gi|7...	ref|NP_060329.1| uridine-cytidine kinase 1-like 1 [Homo sapiens] , gi|7020678|dbj|BAA91230.1| unname...	2	2	4.4%	61140
[View Peptides]	PRKCSH	protein kinase C substrate 80K-H	2	2	4.4%	59296
[View Peptides]	MCM3	minichromosome maintenance complex component 3	2	2	4.3%	90981
[View Peptides]	SMARCC1	SWI/SNF related, matrix associated, actin dependent regulator of chromatin, subfamily c, member 1	3	4	4.3%	122753
[View Peptides]	SPTBN2	spectrin, beta, non-erythrocytic 2	6	6	4.2%	271293
[View Peptides]	MED16	mediator complex subunit 16	2	2	4.2%	96793
[View Peptides]	CDC2L2	cell division cycle 2-like 2 (PITSLRE proteins)	2	2	4.1%	91004
[View Peptides]	EEF1A1	eukaryotic translation elongation factor 1 alpha 1	2	2	4.1%	50141
[View Peptides]	EEF1A2	eukaryotic translation elongation factor 1 alpha 2	2	2	4.1%	50470
[View Peptides]	COPB2	coatomer protein complex, subunit beta 2 (beta prime)	2	2	4.0%	102487
[View Peptides]	MOV10	Mov10, Moloney leukemia virus 10, homolog (mouse)	2	2	4.0%	113671
[View Peptides]	CPSF1	cleavage and polyadenylation specific factor 1, 160kDa	4	6	4.0%	160883
[View Peptides]	CDC2L1	cell division cycle 2-like 1 (PITSLRE proteins)	2	2	4.0%	92707
[View Peptides]	TFRC	transferrin receptor (p90, CD71)	2	3	3.9%	84901
[View Peptides]	PLCB3	phospholipase C, beta 3 (phosphatidylinositol-specific)	3	5	3.9%	138799
[View Peptides]	RAD50	RAD50 homolog (S. cerevisiae)	3	3	3.9%	153892
[View Peptides]	POLE	polymerase (DNA directed), epsilon	5	12	3.9%	261529
[View Peptides]	MED1	mediator complex subunit 1	4	4	3.8%	168437
[View Peptides]	ORC3L	origin recognition complex, subunit 3-like (yeast)	2	2	3.8%	82254
[View Peptides]	POP1	processing of precursor 1, ribonuclease P/MRP subunit (S. cerevisiae)	2	2	3.6%	114709
[View Peptides]	EIF5B	eukaryotic translation initiation factor 5B	3	4	3.6%	138799
[View Peptides]	SMC1A	structural maintenance of chromosomes 1A	3	4	3.6%	143233
[View Peptides]	ACIN1	apoptotic chromatin condensation inducer 1	3	4	3.6%	151887
[View Peptides]	ZNF638	zinc finger protein 638	4	4	3.5%	220623
[View Peptides]	HLTF	helicase-like transcription factor	2	2	3.5%	113928
[View Peptides]	C14orf21	chromosome 14 open reading frame 21	2	3	3.5%	69438
[View Peptides]	PRPF4B	PRP4 pre-mRNA processing factor 4 homolog B (yeast)	2	5	3.4%	116973
[View Peptides]	UBE1	ubiquitin-activating enzyme E1	2	2	3.4%	117849
[View Peptides]	POLD1	polymerase (DNA directed), delta 1, catalytic subunit 125kDa	2	2	3.4%	123631
[View Peptides]	gi|67476986	gi|67476986|sp|Q9BTC0|DIDO1_HUMAN Death-inducer obliterator 1 (DIO-1) (Death-associated transcriptio...	3	3	3.4%	129150
[View Peptides]	ADAR	adenosine deaminase, RNA-specific	2	3	3.4%	135995

[View Peptides]	RIF1	RAP1 interacting factor homolog (yeast)	5	5	3.3%	274464
[View Peptides]	NOL14	nucleolar protein 14	2	2	3.3%	97668
[View Peptides]	ARHGEF15	Rho guanine nucleotide exchange factor (GEF) 15	2	2	3.3%	91950
[View Peptides]	SMARCA5	SWI/SNF related, matrix associated, actin dependent regulator of chromatin, subfamily a, member 5	2	3	3.2%	121905
[View Peptides]	DCTN1	dynactin 1 (p150, glued homolog, Drosophila)	2	3	3.2%	141694
[View Peptides]	UBTF	upstream binding transcription factor, RNA polymerase I	2	3	3.1%	89406
[View Peptides]	KRT77	keratin 77	2	4	3.1%	61688
[View Peptides]	CHD7	chromodomain helicase DNA binding protein 7	4	4	3.0%	252482
[View Peptides]	BAT2	HLA-B associated transcript 2	4	4	3.0%	228858
[View Peptides]	ATXN2L	ataxin 2-like	2	2	3.0%	113374
[View Peptides]	POLR1B	polymerase (RNA) I polypeptide B, 128kDa	2	3	2.9%	122138
[View Peptides]	RRP12	ribosomal RNA processing 12 homolog (S. cerevisiae)	2	3	2.9%	143702
[View Peptides]	CHD3	chromodomain helicase DNA binding protein 3	3	3	2.7%	220690
[View Peptides]	PDCD11	programmed cell death 11	3	4	2.7%	208733
[View Peptides]	STAG2	stromal antigen 2	2	2	2.7%	141326
[View Peptides]	TMF1	TATA element modulatory factor 1	2	2	2.6%	123171
[View Peptides]	WDR33	WD repeat domain 33	2	3	2.4%	145921
[View Peptides]	VCL	vinculin	2	2	2.4%	123799
[View Peptides]	DNM1	dynamin 1	2	2	2.3%	97407
[View Peptides]	DNM2	dynamin 2	2	2	2.3%	98064
[View Peptides]	PCF11	PCF11, cleavage and polyadenylation factor subunit, homolog (S. cerevisiae)	3	3	2.3%	183980
[View Peptides]	TATDN2	TatD DNase domain containing 2	2	2	2.2%	85039
[View Peptides]	PBRM1	polybromo 1	2	2	2.1%	192946
[View Peptides]	MLLT4	myeloid/lymphoid or mixed-lineage leukemia (trithorax homolog, Drosophila); translocated to, 4	2	2	1.8%	205604
[View Peptides]	RRBP1	ribosome binding protein 1 homolog 180kDa (dog)	2	2	1.8%	152472
[View Peptides]	THOC2	THO complex 2	2	3	1.7%	169581
[View Peptides]	TRRAP	transformation/transcription domain-associated protein	4	4	1.7%	437589
[View Peptides]	BAZ1A	bromodomain adjacent to zinc finger domain, 1A	2	2	1.6%	178674
[View Peptides]	UTP20	UTP20, small subunit (SSU) processome component, homolog (yeast)	2	2	1.5%	318426
[View Peptides]	GCN1L1	GCN1 general control of amino-acid synthesis 1-like 1 (yeast)	2	3	1.5%	292742
[View Peptides]	NIPBL	Nipped-B homolog (Drosophila)	2	2	1.4%	316051
[View Peptides]	HCFC1	host cell factor C1 (VP16-accessory protein)	2	2	1.4%	208840
[View Peptides]	GTF3C1	general transcription factor IIIC, polypeptide 1, alpha 220kDa	2	3	1.2%	238293
[View Peptides]	ASCC3	activating signal cointegrator 1 complex subunit 3	2	2	1.0%	251488
[View Peptides]	EPPK1	epiplakin 1	2	2	0.8%	553102

[View Peptides]	DYNC1H1	dynein, cytoplasmic 1, heavy chain 1	2	2	0.7%	532412
[View Peptides]	PLEC1	plectin 1, intermediate filament binding protein 500kDa	2	3	0.7%	531742

YRC Informatics Platform - Version 3.0
Created and Maintained by: Michael Riffle

View Mass Spectrometry Run

Run Information:

Organism: *Homo sapiens*
Run Date: 2008-03-11
Project: Mechanism of alternative splicing of human insulin receptor
DTA SELECT: Download DTASelect Filter text
Download DTASelect HTML file
Download Unfiltered DTASelect text file
Comments: RNA binding splicing regulatory proteins sample: m1
[Edit Comments]

FILTERED Run Results: [unfilter]

	Hit Protein	Protein Desc	Sequence Count	Spectrum Count	Sequence Coverage	Mol. Wt.
[View Peptides]	ACTG1	actin, gamma 1	34	346	63.2%	41793
[View Peptides]	ACTB	actin, beta	34	346	61.1%	41737
[View Peptides]	NONO	non-POU domain containing, octamer-binding	65	621	58.6%	54232
[View Peptides]	SNRPD2	small nuclear ribonucleoprotein D2 polypeptide 16.5kDa	6	25	52.5%	13527
[View Peptides]	HNRNPA2B1	heterogeneous nuclear ribonucleoprotein A2/B1	16	66	52.1%	37430
[View Peptides]	SEPT2	septin 2	15	77	49.6%	41487
[View Peptides]	CFL1	cofilin 1 (non-muscle)	7	13	48.8%	18502
[View Peptides]	SNRPD3	small nuclear ribonucleoprotein D3 polypeptide 18kDa	5	14	47.6%	13916
[View Peptides]	HNRPH1	heterogeneous nuclear ribonucleoprotein H1 (H)	16	140	45.9%	49229
[View Peptides]	SRP19	signal recognition particle 19kDa	4	6	43.8%	16186
[View Peptides]	PSMA2	proteasome (prosome, macropain) subunit, alpha type, 2	8	11	43.6%	25899
[View Peptides]	GTF2I	general transcription factor II, i	56	392	43.5%	112416
[View Peptides]	RPLP2	ribosomal protein, large, P2	2	2	43.5%	11665
[View Peptides]	PCNA	proliferating cell nuclear antigen	8	17	42.9%	28769

[View Peptides]	GAPDH	glyceraldehyde-3-phosphate dehydrogenase	17	189	42.7%	36053
[View Peptides]	SEPT9	septin 9	25	99	41.8%	65369
[View Peptides]	PARP1	poly (ADP-ribose) polymerase family, member 1	44	175	41.8%	113084
[View Peptides]	RPL30	ribosomal protein L30	3	12	40.9%	12784
[View Peptides]	SFPQ	splicing factor proline/glutamine-rich (polypyrimidine tract binding protein associated)	55	526	40.7%	76150
[View Peptides]	RPLP0	ribosomal protein, large, P0	12	21	40.4%	34274
[View Peptides]	PKM2	pyruvate kinase, muscle	17	43	39.4%	57937
[View Peptides]	SEPT7	septin 7	14	88	39.4%	50680
[View Peptides]	PTBP1	polypyrimidine tract binding protein 1	17	60	38.8%	57221
[View Peptides]	PGK1	phosphoglycerate kinase 1	12	32	38.8%	44615
[View Peptides]	ENO1	enolase 1, (alpha)	15	46	38.7%	47169
[View Peptides]	ACTN4	actinin, alpha 4	28	66	38.2%	104854
[View Peptides]	HNRPH2	heterogeneous nuclear ribonucleoprotein H2 (H')	11	31	38.1%	49264
[View Peptides]	TUBA4A, TUBA1B	tubulin, alpha 1b, tubulin, alpha 4a	12	31	37.3%	50152
[View Peptides]	TUBA1A	tubulin, alpha 1a	12	30	37.3%	50136
[View Peptides]	TARDBP	TAR DNA binding protein	10	18	37.0%	44740
[View Peptides]	SNRPD1	small nuclear ribonucleoprotein D1 polypeptide 16kDa	3	28	37.0%	13282
[View Peptides]	NME2	non-metastatic cells 2, protein (NM23B) expressed in	5	10	36.8%	17298
[View Peptides]	KRT1	keratin 1 (epidermolytic hyperkeratosis)	20	40	35.7%	66018
[View Peptides]	ACTN1	actinin, alpha 1	25	43	35.5%	103058
[View Peptides]	TUBA4A	tubulin, alpha 4a	11	19	35.3%	49924
[View Peptides]	RPL9	ribosomal protein L9	5	14	34.9%	21863
[View Peptides]	ANXA2	annexin A2	10	31	34.2%	38604
[View Peptides]	YWHAE	tyrosine 3-monooxygenase/tryptophan 5-monooxygenase activation protein, epsilon polypeptide	7	18	33.3%	29174
[View Peptides]	SFRS1	splicing factor, arginine/serine-rich 1 (splicing factor 2, alternate splicing factor)	11	89	33.1%	27745
[View Peptides]	HIST1H4L, HIST4H...	histone cluster 2, H4a, histone cluster 1, H4i, histone cluster 1, H4l, histone cluster 1, H4e, hist...	3	4	33.0%	11367
[View Peptides]	NOLA3	nucleolar protein family A, member 3 (H/ACA small nucleolar RNPs)	2	2	32.8%	7706
[View Peptides]	ALDOA	aldolase A, fructose-bisphosphate	11	37	32.7%	39420
[View Peptides]	HSPA8	heat shock 70kDa protein 8	19	38	32.5%	70898
[View Peptides]	EEF2	eukaryotic translation elongation factor 2	26	50	32.5%	95338
[View Peptides]	FLNA	filamin A, alpha (actin binding protein 280)	66	167	32.2%	280759
[View Peptides]	LDHB	lactate dehydrogenase B	9	23	32.0%	36639
[View Peptides]	PPP1CC	protein phosphatase 1, catalytic subunit, gamma isoform	9	92	31.9%	36984

[View Peptides]	SEPT10	septin 10	13	37	31.5%	59982
[View Peptides]	PPP1CA	protein phosphatase 1, catalytic subunit, alpha isoform	9	91	31.5%	37512
[View Peptides]	SYNCRIP	synaptotagmin binding, cytoplasmic RNA interacting protein	14	34	31.3%	69633
[View Peptides]	WDR74	WD repeat domain 74	7	10	31.2%	42441
[View Peptides]	EIF2S1	eukaryotic translation initiation factor 2, subunit 1 alpha, 35kDa	7	8	31.1%	36112
[View Peptides]	THOC4	THO complex 4	5	15	31.1%	26888
[View Peptides]	RPL12	ribosomal protein L12	3	8	30.9%	17819
[View Peptides]	PACSIN3	protein kinase C and casein kinase substrate in neurons 3	13	43	30.7%	48487
[View Peptides]	CCT3	chaperonin containing TCP1, subunit 3 (gamma)	12	18	30.3%	60534
[View Peptides]	SNRPA1	small nuclear ribonucleoprotein polypeptide A'	4	7	30.2%	28416
[View Peptides]	HNRNPL	heterogeneous nuclear ribonucleoprotein L	11	23	30.1%	60187
[View Peptides]	EIF4A1	eukaryotic translation initiation factor 4A, isoform 1	10	18	30.0%	46154
[View Peptides]	SND1	staphylococcal nuclease and tudor domain containing 1	26	94	29.7%	101997
[View Peptides]	PSMB4	proteasome (prosome, macropain) subunit, beta type, 4	4	8	29.5%	29192
[View Peptides]	SF3A3	splicing factor 3a, subunit 3, 60kDa	12	21	29.3%	58849
[View Peptides]	YWHAZ	tyrosine 3-monooxygenase/tryptophan 5-monooxygenase activation protein, zeta polypeptide	6	19	29.0%	27745
[View Peptides]	gi|21619210, gi|...	pir||MOHULP myosin regulatory light chain, placental - human , gi|5453740|ref|NP_006462.1| myosin re...	3	6	28.7%	19794
[View Peptides]	CLIC1	chloride intracellular channel 1	4	9	28.6%	26923
[View Peptides]	RPL36	ribosomal protein L36	4	6	28.6%	12254
[View Peptides]	ACTG2	actin, gamma 2, smooth muscle, enteric	13	49	28.5%	41877
[View Peptides]	ACTC1	actin, alpha, cardiac muscle 1	13	49	28.4%	42019
[View Peptides]	ACTA2	actin, alpha 2, smooth muscle, aorta	13	49	28.4%	42009
[View Peptides]	HMGB1	high-mobility group box 1	6	8	28.4%	24894
[View Peptides]	ACTA1	actin, alpha 1, skeletal muscle	13	49	28.4%	42051
[View Peptides]	CFL2	cofilin 2 (muscle)	4	5	28.3%	18737
[View Peptides]	SUB1	SUB1 homolog (S. cerevisiae)	4	16	28.3%	14395
[View Peptides]	HNRNPR	heterogeneous nuclear ribonucleoprotein R	15	35	28.1%	70943
[View Peptides]	EXOSC7	exosome component 7	6	8	27.8%	31835
[View Peptides]	SPTAN1	spectrin, alpha, non-erythrocytic 1 (alpha-fodrin)	49	135	27.8%	284526
[View Peptides]	HSP90AB1	heat shock protein 90kDa alpha (cytosolic), class B member 1	13	33	27.6%	83264
[View Peptides]	PSMB1	proteasome (prosome, macropain) subunit, beta type, 1	4	10	27.4%	26489
[View Peptides]	PSMA7	proteasome (prosome, macropain) subunit, alpha type, 7	5	6	27.4%	27887
[View Peptides]	EFTUD2	elongation factor Tu GTP binding domain containing 2	19	33	27.0%	109436
[View Peptides]	APOBEC3C	apolipoprotein B mRNA editing enzyme, catalytic polypeptide-like 3C	3	5	26.8%	22826

[View Peptides]	YWHAB	tyrosine 3-monooxygenase/tryptophan 5-monooxygenase activation protein, beta polypeptide	5	9	26.8%	28082
[View Peptides]	CIRBP	cold inducible RNA binding protein	4	16	26.7%	18648
[View Peptides]	VCP	valosin-containing protein	15	38	26.6%	89322
[View Peptides]	EIF5AP1, EIF5A	eukaryotic translation initiation factor 5A pseudogene 1, eukaryotic translation initiation factor 5...	2	3	26.6%	16832
[View Peptides]	TAGLN2	transgelin 2	3	7	26.6%	22391
[View Peptides]	LRPPRC	leucine-rich PPR-motif containing	22	44	26.5%	145201
[View Peptides]	RPS8	ribosomal protein S8	4	8	26.4%	24205
[View Peptides]	PCBP1	poly(rC) binding protein 1	7	13	26.4%	37498
[View Peptides]	SNRPG	small nuclear ribonucleoprotein polypeptide G	4	5	26.3%	8496
[View Peptides]	TPI1	triosephosphate isomerase 1	4	7	26.1%	26669
[View Peptides]	RUVBL1	RuvB-like 1 (E. coli)	7	10	26.1%	50228
[View Peptides]	PPIB	peptidylprolyl isomerase B (cyclophilin B)	6	14	26.0%	22742
[View Peptides]	RAN	RAN, member RAS oncogene family	7	8	25.9%	24423
[View Peptides]	MAP4	microtubule-associated protein 4	24	49	25.8%	121019
[View Peptides]	EMG1	EMG1 nucleolar protein homolog (S. cerevisiae)	5	7	25.8%	26720
[View Peptides]	EIF4H	eukaryotic translation initiation factor 4H	4	17	25.8%	27385
[View Peptides]	IQGAP1	IQ motif containing GTPase activating protein 1	29	55	25.8%	189251
[View Peptides]	CALR	calreticulin	7	16	25.7%	48142
[View Peptides]	PDIA6	protein disulfide isomerase family A, member 6	6	13	25.7%	48121
[View Peptides]	RPL7A	ribosomal protein L7a	7	19	25.6%	29996
[View Peptides]	RPL18	ribosomal protein L18	5	11	25.5%	21634
[View Peptides]	gi|41019521	sp|Q9NR30|DD21_HUMAN Nucleolar RNA helicase II (Nucleolar RNA helicase Gu) (RH II/Gu) (DEAD-box prot...	19	44	25.4%	87357
[View Peptides]	DDX1	DEAD (Asp-Glu-Ala-Asp) box polypeptide 1	12	43	25.4%	82432
[View Peptides]	RPL23	ribosomal protein L23	2	9	25.0%	14865
[View Peptides]	gi|545257, gi|21...	sp|P35527|K1CI_HUMAN Keratin, type I cytoskeletal 9 (Cytokeratin 9) (K9) (CK 9) , gi|545257|gb|AAC60...	11	27	24.9%	61987
[View Peptides]	PGAM1	phosphoglycerate mutase 1 (brain)	5	7	24.8%	28804
[View Peptides]	PSMA6	proteasome (prosome, macropain) subunit, alpha type, 6	4	4	24.8%	27399
[View Peptides]	SEPT11	septin 11	15	42	24.5%	49398
[View Peptides]	RPL4	ribosomal protein L4	11	19	24.4%	47697
[View Peptides]	SEPT6	septin 6	12	29	24.4%	49717
[View Peptides]	DDX5	DEAD (Asp-Glu-Ala-Asp) box polypeptide 5	14	28	24.3%	69148
[View Peptides]	CCT4	chaperonin containing TCP1, subunit 4 (delta)	8	17	24.3%	57924
[View Peptides]	BUB3	BUB3 budding uninhibited by benzimidazoles 3 homolog (yeast)	5	5	24.1%	37155
[View Peptides]	SERBP1	SERPINE1 mRNA binding protein 1	9	19	24.0%	44965

[View Peptides]	HSPA1A, HSPA1B	heat shock 70kDa protein 1B, heat shock 70kDa protein 1A	9	14	23.9%	70052
[View Peptides]	NPM1	nucleophosmin (nucleolar phosphoprotein B23, numatrin)	7	37	23.8%	32575
[View Peptides]	XRN2	5'-3' exoribonuclease 2	15	37	23.7%	108583
[View Peptides]	RPS5	ribosomal protein S5	3	6	23.5%	22876
[View Peptides]	HSPA5	heat shock 70kDa protein 5 (glucose-regulated protein, 78kDa)	12	21	23.2%	72333
[View Peptides]	MSN	moesin	13	26	23.2%	67820
[View Peptides]	CCT7	chaperonin containing TCP1, subunit 7 (eta)	9	22	23.0%	59367
[View Peptides]	DHX15	DEAH (Asp-Glu-Ala-His) box polypeptide 15	12	29	22.8%	90933
[View Peptides]	EIF2S3	eukaryotic translation initiation factor 2, subunit 3 gamma, 52kDa	8	11	22.7%	51110
[View Peptides]	RCC2	regulator of chromosome condensation 2	8	11	22.6%	56085
[View Peptides]	PPP1CB	protein phosphatase 1, catalytic subunit, beta isoform	6	67	22.6%	37187
[View Peptides]	gi|119621172, gi...	gi|9963797|gb|AAG09698.1|AF184213_1 ht006 protein [Homo sapiens], gi|88192892|pdb|2F9D|B Chain B, 2....	2	2	22.4%	14585
[View Peptides]	SPTBN1	spectrin, beta, non-erythrocytic 1	35	64	22.2%	274630
[View Peptides]	M6PRBP1	mannose-6-phosphate receptor binding protein 1	5	7	22.1%	47047
[View Peptides]	CCT2	chaperonin containing TCP1, subunit 2 (beta)	9	19	22.1%	57488
[View Peptides]	PRPF19	PRP19/PSO4 pre-mRNA processing factor 19 homolog (S. cerevisiae)	9	23	22.0%	55181
[View Peptides]	ANXA1	annexin A1	5	9	21.7%	38714
[View Peptides]	PSMA5	proteasome (prosome, macropain) subunit, alpha type, 5	3	3	21.6%	26411
[View Peptides]	ARPC1B	actin related protein 2/3 complex, subunit 1B, 41kDa	5	9	21.5%	40950
[View Peptides]	ANXA5	annexin A5	5	8	21.2%	35937
[View Peptides]	LDHA	lactate dehydrogenase A	5	13	20.8%	36689
[View Peptides]	DDX39	DEAD (Asp-Glu-Ala-Asp) box polypeptide 39	9	19	20.8%	49130
[View Peptides]	DDX17	DEAD (Asp-Glu-Ala-Asp) box polypeptide 17	12	23	20.8%	72372
[View Peptides]	TARS	threonyl-tRNA synthetase	10	17	20.7%	83435
[View Peptides]	gi|59797947, gi|...	gi|59797947|sp|Q8IUH3|DRBP1_HUMAN Developmentally-regulated RNA-binding protein 1 (RB-1), gi|2283132...	6	9	20.4%	53503
[View Peptides]	EXOSC8	exosome component 8	3	5	20.3%	30040
[View Peptides]	HNRNPA1	heterogeneous nuclear ribonucleoprotein A1	9	25	20.2%	38846
[View Peptides]	U2AF2	U2 small nuclear RNA auxiliary factor 2	5	14	20.2%	53501
[View Peptides]	RPS6	ribosomal protein S6	4	5	20.1%	28681
[View Peptides]	WDR5	WD repeat domain 5	5	6	20.1%	36589
[View Peptides]	RFC4	replication factor C (activator 1) 4, 37kDa	6	8	20.1%	39682
[View Peptides]	EEF1A1	eukaryotic translation elongation factor 1 alpha 1	10	18	20.1%	50141

[View Peptides]	KRT10	keratin 10 (epidermolytic hyperkeratosis; keratosis palmaris et plantaris)	10	25	20.1%	59519
[View Peptides]	HNRPM	heterogeneous nuclear ribonucleoprotein M	12	17	20.1%	77516
[View Peptides]	TCP1	t-complex 1	8	21	20.0%	60344
[View Peptides]	WDR3	WD repeat domain 3	12	16	19.9%	106099
[View Peptides]	RPL18A	ribosomal protein L18a	3	5	19.9%	20762
[View Peptides]	HNRPA3	heterogeneous nuclear ribonucleoprotein A3	6	12	19.8%	39595
[View Peptides]	TUBB	tubulin, beta	7	18	19.8%	49671
[View Peptides]	DHX9	DEAH (Asp-Glu-Ala-His) box polypeptide 9	19	49	19.8%	140881
[View Peptides]	CKB	creatine kinase, brain	4	7	19.7%	42644
[View Peptides]	DENR	density-regulated protein	2	2	19.7%	22092
[View Peptides]	gi|230867, gi|23...	gi|230868|pdb|3GPD|G Chain G, Twinning In Crystals Of Human Skeletal Muscle D- Glyceraldehyde-3-Phos...	8	17	19.5%	35876
[View Peptides]	PCBP2	poly(rC) binding protein 2	5	6	19.5%	38580
[View Peptides]	TIAL1	TIA1 cytotoxic granule-associated RNA binding protein-like 1	5	12	19.5%	41591
[View Peptides]	TRIM28	tripartite motif-containing 28	9	21	19.5%	88550
[View Peptides]	FLNB	filamin B, beta (actin binding protein 278)	32	65	19.4%	278193
[View Peptides]	HSP90B1	heat shock protein 90kDa beta (Grp94), member 1	12	16	19.3%	92469
[View Peptides]	VIM	vimentin	6	10	19.3%	53652
[View Peptides]	RPS20	ribosomal protein S20	2	2	19.3%	13373
[View Peptides]	PSMB5	proteasome (prosome, macropain) subunit, beta type, 5	3	3	19.2%	22897
[View Peptides]	CCT8	chaperonin containing TCP1, subunit 8 (theta)	8	18	19.2%	59621
[View Peptides]	CDC5L	CDC5 cell division cycle 5-like (S. pombe)	10	19	19.1%	92251
[View Peptides]	RFC5	replication factor C (activator 1) 5, 36.5kDa	4	7	19.1%	38497
[View Peptides]	RUVBL2	RuvB-like 2 (E. coli)	7	12	19.0%	51157
[View Peptides]	HNRPK	heterogeneous nuclear ribonucleoprotein K	7	16	19.0%	50976
[View Peptides]	DDB2	damage-specific DNA binding protein 2, 48kDa	5	11	18.7%	47864
[View Peptides]	BAT1	HLA-B associated transcript 1	8	24	18.7%	48991
[View Peptides]	gi|6469388, gi|2...	emb|CAB61788.1| dJ18C9.3 (novel protein similar to high mobility group protein 2A (HMG2A)) [Homo sa...	3	3	18.6%	20854
[View Peptides]	G3BP1	GTPase activating protein (SH3 domain) binding protein 1	7	16	18.5%	52164
[View Peptides]	ALDOC	aldolase C, fructose-bisphosphate	5	20	18.4%	39456
[View Peptides]	PRKDC	protein kinase, DNA-activated, catalytic polypeptide	55	161	18.3%	469093
[View Peptides]	SF3A1	splicing factor 3a, subunit 1, 120kDa	11	23	18.2%	88886
[View Peptides]	GSTM3	glutathione S-transferase M3 (brain)	3	5	18.2%	26560
[View Peptides]	CSNK2B	casein kinase 2, beta polypeptide	2	5	18.1%	24942
[View Peptides]	RFC2	replication factor C (activator 1) 2, 40kDa	4	9	18.1%	39157
[View Peptides]	CCT6A	chaperonin containing TCP1, subunit 6A (zeta 1)	8	20	18.1%	58024

[View Peptides]	XRCC6	X-ray repair complementing defective repair in Chinese hamster cells 6 (Ku autoantigen, 70kDa)	7	18	18.1%	69843
[View Peptides]	RPL11	ribosomal protein L11	2	4	18.0%	20252
[View Peptides]	gi|40254816, gi|...	gi|40254816|ref|NP_005339.2| heat shock protein 90kDa alpha (cytosolic), class A member 1 isoform 2 ...	10	24	17.9%	84674
[View Peptides]	YWHAH	tyrosine 3-monooxygenase/tryptophan 5-monooxygenase activation protein, eta polypeptide	4	5	17.9%	28219
[View Peptides]	EXOSC2	exosome component 2	3	7	17.7%	32789
[View Peptides]	SFRS10	splicing factor, arginine/serine-rich 10 (transformer 2 homolog, Drosophila)	5	34	17.7%	33666
[View Peptides]	gi|119717, gi|48...	pir||A34400 ezrin [validated] - human , gi|31283|emb|CAA35893.1| unnamed protein product [Homo sapie...	10	18	17.7%	69399
[View Peptides]	SRP14	signal recognition particle 14kDa (homologous Alu RNA binding protein)	2	7	17.6%	14544
[View Peptides]	GSTP1	glutathione S-transferase pi	2	2	17.6%	23356
[View Peptides]	FEN1	flap structure-specific endonuclease 1	5	11	17.6%	42593
[View Peptides]	KRT6A	keratin 6A	9	11	17.6%	60045
[View Peptides]	gi|2119222, gi|9...	gi|908801|gb|AAC41769.1| keratin type II, pir||I61768 keratin 6c, type II - human, gi|17505189|ref|N...	9	11	17.6%	60200
[View Peptides]	NARS	asparaginyl-tRNA synthetase	6	10	17.5%	62943
[View Peptides]	ANP32E	acidic (leucine-rich) nuclear phosphoprotein 32 family, member E	3	5	17.5%	30692
[View Peptides]	HDGF	hepatoma-derived growth factor (high-mobility group protein 1-like)	3	10	17.5%	26788
[View Peptides]	RPL23A	ribosomal protein L23a	2	3	17.3%	17695
[View Peptides]	KCTD14	potassium channel tetramerisation domain containing 14	3	5	17.3%	26188
[View Peptides]	CORO1C	coronin, actin binding protein, 1C	7	14	17.3%	53249
[View Peptides]	WDR36	WD repeat domain 36	11	16	17.2%	105322
[View Peptides]	KPNA2	karyopherin alpha 2 (RAG cohort 1, importin alpha 1)	5	9	17.2%	57862
[View Peptides]	SFRS7	splicing factor, arginine/serine-rich 7, 35kDa	5	21	17.2%	27367
[View Peptides]	NIP7	nuclear import 7 homolog (S. cerevisiae)	2	2	17.2%	20463
[View Peptides]	PFN1	profilin 1	2	3	17.1%	15054
[View Peptides]	POLR1C	polymerase (RNA) I polypeptide C, 30kDa	4	9	17.1%	39250
[View Peptides]	TUBB2C	tubulin, beta 2C	6	10	17.1%	49831
[View Peptides]	TUBB3, TUBB4	tubulin, beta 4, tubulin, beta 3	6	12	16.9%	50433
[View Peptides]	gi|51338655	gi|51338655|sp|P62861|RS30_HUMAN 40S ribosomal protein S30	2	2	16.9%	6648
[View Peptides]	C14orf166	chromosome 14 open reading frame 166	3	5	16.8%	28068
[View Peptides]	EIF2S2	eukaryotic translation initiation factor 2, subunit 2 beta, 38kDa	3	4	16.8%	38388
[View Peptides]	PA2G4	proliferation-associated 2G4, 38kDa	6	9	16.8%	43787
[View Peptides]	EIF4A3	eukaryotic translation initiation factor 4A, isoform 3	4	5	16.8%	46871

[View Peptides]	HDAC1	histone deacetylase 1	5	7	16.8%	55103
[View Peptides]	SFRS9	splicing factor, arginine/serine-rich 9	4	16	16.7%	25542
[View Peptides]	ATIC	5-aminoimidazole-4-carboxamide ribonucleotide formyltransferase/IMP cyclohydrolase	5	10	16.6%	64616
[View Peptides]	RPL10A	ribosomal protein L10a	3	12	16.6%	24831
[View Peptides]	DSTN	destrin (actin depolymerizing factor)	2	2	16.4%	18506
[View Peptides]	RAB1B	RAB1B, member RAS oncogene family	2	2	16.4%	22171
[View Peptides]	EEF1B2	eukaryotic translation elongation factor 1 beta 2	3	7	16.4%	24764
[View Peptides]	TKT	transketolase (Wernicke-Korsakoff syndrome)	6	12	16.4%	67878
[View Peptides]	FKBP4	FK506 binding protein 4, 59kDa	5	5	16.3%	51805
[View Peptides]	ACTL6A	actin-like 6A	5	6	16.3%	47461
[View Peptides]	POLDIP3	polymerase (DNA-directed), delta interacting protein 3	4	8	16.2%	46089
[View Peptides]	RAB1A	RAB1A, member RAS oncogene family	2	3	16.1%	22678
[View Peptides]	VDAC2	voltage-dependent anion channel 2	3	4	16.1%	38093
[View Peptides]	HSPD1	heat shock 60kDa protein 1 (chaperonin)	6	11	16.1%	61055
[View Peptides]	PDIA4	protein disulfide isomerase family A, member 4	6	12	16.1%	72933
[View Peptides]	TPD52L2	tumor protein D52-like 2	2	2	16.0%	22238
[View Peptides]	RPS3	ribosomal protein S3	3	5	16.0%	26688
[View Peptides]	HSPA4	heat shock 70kDa protein 4	8	15	16.0%	94300
[View Peptides]	TOP2A	topoisomerase (DNA) II alpha 170kDa	17	34	15.9%	174384
[View Peptides]	EWSR1	Ewing sarcoma breakpoint region 1	14	48	15.9%	68478
[View Peptides]	SFRS3	splicing factor, arginine/serine-rich 3	3	17	15.9%	19330
[View Peptides]	RBM39	RNA binding motif protein 39	8	13	15.8%	59380
[View Peptides]	TPT1	tumor protein, translationally-controlled 1	2	3	15.7%	19595
[View Peptides]	APEX1	APEX nuclease (multifunctional DNA repair enzyme) 1	4	5	15.7%	35554
[View Peptides]	METAP2	methionyl aminopeptidase 2	4	5	15.7%	52892
[View Peptides]	MORC2	MORC family CW-type zinc finger 2	11	16	15.7%	110724
[View Peptides]	PRDX6	peroxiredoxin 6	2	4	15.6%	25035
[View Peptides]	HIST1H1C	histone cluster 1, H1c	7	16	15.5%	21365
[View Peptides]	BLVRB	biliverdin reductase B (flavin reductase (NADPH))	2	2	15.5%	22119
[View Peptides]	RPL14	ribosomal protein L14	3	7	15.5%	23290
[View Peptides]	EIF6	eukaryotic translation initiation factor 6	3	6	15.5%	26599
[View Peptides]	TIA1	TIA1 cytotoxic granule-associated RNA binding protein	4	9	15.5%	42961
[View Peptides]	ASCC3L1	activating signal cointegrator 1 complex subunit 3-like 1	24	50	15.4%	244505
[View Peptides]	DNAJC9	DnaJ (Hsp40) homolog, subfamily C, member 9	2	3	15.4%	29910
[View Peptides]	HNRNPU	heterogeneous nuclear ribonucleoprotein U (scaffold attachment factor A)	11	18	15.3%	90480
[View Peptides]	SERPINB1	serpin peptidase inhibitor, clade B (ovalbumin), member 1	4	9	15.3%	42742

[View Peptides]	PSMA4	proteasome (prosome, macropain) subunit, alpha type, 4	2	4	15.3%	29484
[View Peptides]	SEC22B	SEC22 vesicle trafficking protein homolog B (S. cerevisiae)	2	2	15.3%	24741
[View Peptides]	CNBP	CCHC-type zinc finger, nucleic acid binding protein	2	4	15.3%	19463
[View Peptides]	CKAP5	cytoskeleton associated protein 5	24	47	15.2%	225507
[View Peptides]	NOL1	nucleolar protein 1, 120kDa	10	13	15.1%	94078
[View Peptides]	HIST1H1E	histone cluster 1, H1e	7	16	15.1%	21865
[View Peptides]	ASS1	argininosuccinate synthetase 1	4	38	15.0%	46530
[View Peptides]	SMS	spermine synthase	3	4	15.0%	41268
[View Peptides]	EXOSC5	exosome component 5	2	3	14.9%	25249
[View Peptides]	HNRPD	heterogeneous nuclear ribonucleoprotein D (AU-rich element RNA binding protein 1, 37kDa)	4	9	14.9%	38434
[View Peptides]	KHDRBS1	KH domain containing, RNA binding, signal transduction associated 1	4	7	14.9%	48227
[View Peptides]	SAFB	scaffold attachment factor B	12	20	14.9%	102640
[View Peptides]	HIST1H1D	histone cluster 1, H1d	7	16	14.9%	22350
[View Peptides]	POLR2E	polymerase (RNA) II (DNA directed) polypeptide E, 25kDa	2	3	14.8%	24611
[View Peptides]	PAICS	phosphoribosylaminoimidazole carboxylase, phosphoribosylaminoimidazole succinocarboxamide synthetase	4	10	14.8%	47079
[View Peptides]	FUS	fusion (involved in t(12;16) in malignant liposarcoma)	14	103	14.8%	53426
[View Peptides]	HDAC2	histone deacetylase 2	4	7	14.8%	55364
[View Peptides]	RPL15	ribosomal protein L15	4	7	14.7%	24146
[View Peptides]	SNRPB2	small nuclear ribonucleoprotein polypeptide B''	3	5	14.7%	25486
[View Peptides]	BCAS2	breast carcinoma amplified sequence 2	2	2	14.7%	26131
[View Peptides]	ANXA4	annexin A4	3	3	14.7%	35883
[View Peptides]	PDIA3	protein disulfide isomerase family A, member 3	6	9	14.7%	56782
[View Peptides]	UBE1	ubiquitin-activating enzyme E1	11	22	14.7%	117849
[View Peptides]	HSPA9	heat shock 70kDa protein 9 (mortalin)	6	9	14.6%	73681
[View Peptides]	MAPRE1	microtubule-associated protein, RP/EB family, member 1	2	2	14.6%	29999
[View Peptides]	PSMB3	proteasome (prosome, macropain) subunit, beta type, 3	2	2	14.6%	22949
[View Peptides]	RPL35A	ribosomal protein L35a	2	3	14.5%	12538
[View Peptides]	RPL10	ribosomal protein L10	2	2	14.5%	24577
[View Peptides]	TUBG2	tubulin, gamma 2	4	7	14.4%	51092
[View Peptides]	TUBG1	tubulin, gamma 1	4	7	14.4%	51170
[View Peptides]	KCNAB2	potassium voltage-gated channel, shaker-related subfamily, beta member 2	5	7	14.4%	41000
[View Peptides]	HYOU1	hypoxia up-regulated 1	8	11	14.1%	111335
[View Peptides]	SFRS5	splicing factor, arginine/serine-rich 5	2	2	14.0%	31264

[View Peptides]	FBL	fibrillarin	3	4	14.0%	33784
[View Peptides]	SAFB2	scaffold attachment factor B2	11	18	14.0%	107474
[View Peptides]	RPS14	ribosomal protein S14	3	4	13.9%	16273
[View Peptides]	RBMX	RNA binding motif protein, X-linked	4	11	13.8%	42332
[View Peptides]	ATP5B	ATP synthase, H+ transporting, mitochondrial F1 complex, beta polypeptide	5	7	13.8%	56560
[View Peptides]	GPI	glucose phosphate isomerase	6	16	13.8%	63147
[View Peptides]	TFRC	transferrin receptor (p90, CD71)	7	7	13.7%	84901
[View Peptides]	XRCC5	X-ray repair complementing defective repair in Chinese hamster cells 5 (double-strand-break rejoinin...	6	19	13.7%	82705
[View Peptides]	DBN1	drebrin 1	5	12	13.7%	71425
[View Peptides]	EBNA1BP2	EBNA1 binding protein 2	3	5	13.7%	34820
[View Peptides]	SRM	spermidine synthase	2	2	13.6%	33825
[View Peptides]	NACA	nascent polypeptide-associated complex alpha subunit	2	2	13.5%	23384
[View Peptides]	API5	apoptosis inhibitor 5	5	9	13.5%	57561
[View Peptides]	ACLY	ATP citrate lyase	11	15	13.5%	120825
[View Peptides]	RPL24	ribosomal protein L24	2	2	13.4%	17779
[View Peptides]	TPM3	tropomyosin 3	5	7	13.4%	32819
[View Peptides]	RBBP4	retinoblastoma binding protein 4	5	7	13.4%	47656
[View Peptides]	WARS	tryptophanyl-tRNA synthetase	4	4	13.4%	53165
[View Peptides]	UGDH	UDP-glucose dehydrogenase	4	5	13.4%	55024
[View Peptides]	EXOSC10	exosome component 10	8	12	13.4%	100831
[View Peptides]	RPS2	ribosomal protein S2	3	4	13.3%	31324
[View Peptides]	RPL8	ribosomal protein L8	4	15	13.2%	28025
[View Peptides]	ECHS1	enoyl Coenzyme A hydratase, short chain, 1, mitochondrial	2	4	13.1%	31387
[View Peptides]	PRDX1	peroxiredoxin 1	2	3	13.1%	22110
[View Peptides]	EEF1G	eukaryotic translation elongation factor 1 gamma	3	4	13.0%	50119
[View Peptides]	RPS4X	ribosomal protein S4, X-linked	2	4	12.9%	29598
[View Peptides]	ENO2	enolase 2 (gamma, neuronal)	3	11	12.9%	47269
[View Peptides]	SET	SET translocation (myeloid leukemia-associated)	3	9	12.8%	33489
[View Peptides]	ILF2	interleukin enhancer binding factor 2, 45kDa	3	4	12.8%	43062
[View Peptides]	SNRP70	small nuclear ribonucleoprotein 70kDa polypeptide (RNP antigen)	5	8	12.8%	51557
[View Peptides]	TCOF1	Treacher Collins-Franceschetti syndrome 1	13	16	12.8%	144312
[View Peptides]	POLR1A	polymerase (RNA) I polypeptide A, 194kDa	13	25	12.8%	194190
[View Peptides]	G3BP2	GTPase activating protein (SH3 domain) binding protein 2	3	8	12.7%	54111
[View Peptides]	PSMD12	proteasome (prosome, macropain) 26S subunit, non-ATPase, 12	4	5	12.7%	52904
[View Peptides]	HNRNPC	heterogeneous nuclear ribonucleoprotein C (C1/C2)	3	5	12.7%	33688

[View Peptides]	GPR137, CAPRIN1	cell cycle associated protein 1, G protein-coupled receptor 137	8	14	12.6%	72752
[View Peptides]	PRPF40A	PRP40 pre-mRNA processing factor 40 homolog A (S. cerevisiae)	8	12	12.6%	108805
[View Peptides]	RPL27	ribosomal protein L27	3	5	12.5%	15798
[View Peptides]	SNRPB	small nuclear ribonucleoprotein polypeptides B and B1	4	15	12.5%	24610
[View Peptides]	SNRPN, SNURF	SNRPN upstream reading frame, small nuclear ribonucleoprotein polypeptide N	4	15	12.5%	24614
[View Peptides]	TPM4	tropomyosin 4	4	9	12.5%	28522
[View Peptides]	gi|550013, gi|10...	gi|550013|gb|AAA85654.1| ribosomal protein L5, pir||S55912 ribosomal protein L5, cytosolic - human ,...	3	3	12.5%	34448
[View Peptides]	TOP1	topoisomerase (DNA) I	9	18	12.5%	90726
[View Peptides]	PRPF8	PRP8 pre-mRNA processing factor 8 homolog (S. cerevisiae)	20	42	12.5%	273599
[View Peptides]	PSMB2	proteasome (prosome, macropain) subunit, beta type, 2	2	3	12.4%	22836
[View Peptides]	C1QBP	complement component 1, q subcomponent binding protein	2	5	12.4%	31362
[View Peptides]	gi|1256837, gi|1...	gi|9558733|ref|NP_037425.1| transformer-2 alpha [Homo sapiens], gi|4033480|sp|Q13595|TRA2A_HUMAN Tra...	2	8	12.4%	32689
[View Peptides]	AKR1C4	aldo-keto reductase family 1, member C4 (chlordecone reductase; 3-alpha hydroxysteroid dehydrogenase...	3	8	12.4%	37095
[View Peptides]	KRT2	keratin 2 (epidermal ichthyosis bullosa of Siemens)	8	12	12.4%	65865
[View Peptides]	GNB2L1	guanine nucleotide binding protein (G protein), beta polypeptide 2-like 1	3	4	12.3%	35077
[View Peptides]	LSM14A	LSM14A, SCD6 homolog A (S. cerevisiae)	5	10	12.3%	50530
[View Peptides]	POLR1E	polymerase (RNA) I polypeptide E, 53kDa	4	5	12.3%	53962
[View Peptides]	SF3B3	splicing factor 3b, subunit 3, 130kDa	11	24	12.3%	135592
[View Peptides]	gi|107222, gi|48...	ref|NP_005372.1| nucleolin [Homo sapiens] , gi|189306|gb|AAA59954.1| nucleolin, gi|128841|sp|P19338|...	8	15	12.2%	76344
[View Peptides]	PSMA3	proteasome (prosome, macropain) subunit, alpha type, 3	3	3	12.2%	28433
[View Peptides]	NP	nucleoside phosphorylase	2	3	12.1%	32148
[View Peptides]	RFC3	replication factor C (activator 1) 3, 38kDa	3	4	12.1%	40556
[View Peptides]	OLA1	Obg-like ATPase 1	3	3	12.1%	44744
[View Peptides]	gi|35909, gi|471...	gi|56206088|emb|CAI22287.1| regulator of chromosome condensation 1 [Homo sapiens], gi|47115323|emb|C...	3	6	12.1%	44969
[View Peptides]	GDI2	GDP dissociation inhibitor 2	4	4	12.1%	50663
[View Peptides]	CPS1	carbamoyl-phosphate synthetase 1, mitochondrial	13	16	12.1%	164939
[View Peptides]	PAK1IP1	PAK1 interacting protein 1	3	4	12.0%	43964
[View Peptides]	NAP1L4	nucleosome assembly protein 1-like 4	2	3	12.0%	42823

[View Peptides]	YWHAQ	tyrosine 3-monooxygenase/tryptophan 5-monooxygenase activation protein, theta polypeptide	3	6	11.8%	27764
[View Peptides]	DAZAP1	DAZ associated protein 1	3	5	11.8%	43383
[View Peptides]	CCT5	chaperonin containing TCP1, subunit 5 (epsilon)	5	8	11.8%	59671
[View Peptides]	TMPO	thymopoietin	4	9	11.8%	75492
[View Peptides]	GMPS	guanine monphosphate synthetase	5	7	11.7%	76715
[View Peptides]	YWHAG	tyrosine 3-monooxygenase/tryptophan 5-monooxygenase activation protein, gamma polypeptide	3	7	11.7%	28303
[View Peptides]	ANP32B	acidic (leucine-rich) nuclear phosphoprotein 32 family, member B	2	6	11.6%	28788
[View Peptides]	ANP32A	acidic (leucine-rich) nuclear phosphoprotein 32 family, member A	2	6	11.6%	28585
[View Peptides]	POLR2C	polymerase (RNA) II (DNA directed) polypeptide C, 33kDa	2	2	11.6%	31441
[View Peptides]	SF3B2	splicing factor 3b, subunit 2, 145kDa	6	8	11.6%	97657
[View Peptides]	KIF23	kinesin family member 23	6	7	11.6%	98105
[View Peptides]	CAND1	cullin-associated and neddylation-dissociated 1	9	11	11.5%	136375
[View Peptides]	MYH9	myosin, heavy chain 9, non-muscle	13	20	11.4%	226530
[View Peptides]	RCN1	reticulocalbin 1, EF-hand calcium binding domain	2	3	11.2%	38890
[View Peptides]	HLA-A	major histocompatibility complex, class I, A	3	3	11.2%	40909
[View Peptides]	PWP2	PWP2 periodic tryptophan protein homolog (yeast)	7	13	11.2%	102451
[View Peptides]	UBAP2L	ubiquitin associated protein 2-like	7	7	11.2%	103930
[View Peptides]	YBX1	Y box binding protein 1	2	2	11.1%	35924
[View Peptides]	RPL6	ribosomal protein L6	3	6	11.1%	32728
[View Peptides]	DNAJC8	DnaJ (Hsp40) homolog, subfamily C, member 8	2	2	11.1%	29842
[View Peptides]	GTF2H3	general transcription factor IIH, polypeptide 3, 34kDa	2	2	11.0%	34378
[View Peptides]	THOC1	THO complex 1	5	9	11.0%	75666
[View Peptides]	HSPA1L	heat shock 70kDa protein 1-like	6	8	10.9%	70375
[View Peptides]	CCT6B	chaperonin containing TCP1, subunit 6B (zeta 2)	3	5	10.9%	57765
[View Peptides]	SFRS2	splicing factor, arginine/serine-rich 2	2	3	10.9%	25476
[View Peptides]	CDC2	cell division cycle 2, G1 to S and G2 to M	2	3	10.8%	34095
[View Peptides]	AKR1C3	aldo-keto reductase family 1, member C3 (3-alpha hydroxysteroid dehydrogenase, type II)	2	6	10.8%	36844
[View Peptides]	THOC3	THO complex 3	3	3	10.8%	38772
[View Peptides]	MAPK1	mitogen-activated protein kinase 1	2	2	10.8%	41390
[View Peptides]	SRP68	signal recognition particle 68kDa	4	6	10.7%	70730
[View Peptides]	PSMC3	proteasome (prosome, macropain) 26S subunit, ATPase, 3	3	5	10.7%	49204
[View Peptides]	SORD	sorbitol dehydrogenase	2	2	10.6%	38297
[View Peptides]	PSD4, PSMD4	proteasome (prosome, macropain) 26S subunit, non-ATPase, 4, pleckstrin and Sec7 domain containing 4	2	2	10.6%	40737

[View Peptides]	HNRPF	heterogeneous nuclear ribonucleoprotein F	4	93	10.6%	45672
[View Peptides]	EIF4A2	eukaryotic translation initiation factor 4A, isoform 2	4	6	10.6%	46402
[View Peptides]	PGD	phosphogluconate dehydrogenase	3	6	10.6%	53140
[View Peptides]	RPA1	replication protein A1, 70kDa	4	5	10.6%	68138
[View Peptides]	PABPN1	poly(A) binding protein, nuclear 1	2	5	10.5%	32749
[View Peptides]	SFRS6	splicing factor, arginine/serine-rich 6	3	4	10.5%	39587
[View Peptides]	RDX	radixin	8	13	10.5%	68564
[View Peptides]	SF3B1	splicing factor 3b, subunit 1, 155kDa	7	18	10.4%	145815
[View Peptides]	VIL1	villin 1	6	7	10.4%	92695
[View Peptides]	gi|2506774	gi|2506774|sp|P05787|K2C8_HUMAN Keratin, type II cytoskeletal 8 (Cytokeratin-8) (CK-8) (Keraton-8) (...	4	5	10.4%	53674
[View Peptides]	EXOSC9	exosome component 9	3	4	10.4%	46978
[View Peptides]	PLRG1	pleiotropic regulator 1 (PRL1 homolog, Arabidopsis)	4	6	10.3%	57194
[View Peptides]	TOP2B	topoisomerase (DNA) II beta 180kDa	12	15	10.3%	183266
[View Peptides]	KIF2C	kinesin family member 2C	4	4	10.2%	81313
[View Peptides]	PPP2R1A	protein phosphatase 2 (formerly 2A), regulatory subunit A , alpha isoform	4	5	10.2%	65224
[View Peptides]	C20orf4	chromosome 20 open reading frame 4	2	2	10.2%	43472
[View Peptides]	PSMB7	proteasome (prosome, macropain) subunit, beta type, 7	2	5	10.1%	29965
[View Peptides]	CDC37	cell division cycle 37 homolog (S. cerevisiae)	2	3	10.1%	44468
[View Peptides]	IFI16	interferon, gamma-inducible protein 16	5	9	10.1%	88275
[View Peptides]	DDX23	DEAD (Asp-Glu-Ala-Asp) box polypeptide 23	6	9	10.1%	95647
[View Peptides]	MBD3	methyl-CpG binding domain protein 3	2	3	10.0%	32844
[View Peptides]	ARPC2	actin related protein 2/3 complex, subunit 2, 34kDa	3	3	10.0%	34333
[View Peptides]	ACTR3	ARP3 actin-related protein 3 homolog (yeast)	2	6	10.0%	47371
[View Peptides]	KRT5	keratin 5 (epidermolysis bullosa simplex, Dowling-Meara/Kobner/Weber-Cockayne types)	6	8	10.0%	62447
[View Peptides]	NAT10	N-acetyltransferase 10	6	6	10.0%	115704
[View Peptides]	SMARCD1	SWI/SNF related, matrix associated, actin dependent regulator of chromatin, subfamily d, member 1	3	5	9.9%	54945
[View Peptides]	RPL3	ribosomal protein L3	2	5	9.9%	46109
[View Peptides]	RRS1	RRS1 ribosome biogenesis regulator homolog (S. cerevisiae)	2	2	9.9%	41193
[View Peptides]	POLB	polymerase (DNA directed), beta	2	3	9.9%	38178
[View Peptides]	SNRPA	small nuclear ribonucleoprotein polypeptide A	3	4	9.9%	31280
[View Peptides]	SEPT5	septin 5	2	6	9.8%	42777
[View Peptides]	EIF4B	eukaryotic translation initiation factor 4B	3	8	9.8%	69224
[View Peptides]	LIG3	ligase III, DNA, ATP-dependent	8	9	9.8%	102691
[View Peptides]	SPTBN2	spectrin, beta, non-erythrocytic 2	14	18	9.7%	271293
[View Peptides]	DDX3X	DEAD (Asp-Glu-Ala-Asp) box polypeptide 3, X-linked	5	10	9.7%	73244

[View Peptides]	PPP2R1B	protein phosphatase 2 (formerly 2A), regulatory subunit A, beta isoform	4	4	9.7%	66202
[View Peptides]	CBR1	carbonyl reductase 1	2	2	9.7%	30375
[View Peptides]	PRDX4	peroxiredoxin 4	2	3	9.6%	30540
[View Peptides]	TYMS	thymidylate synthetase	2	2	9.6%	35716
[View Peptides]	DEK	DEK oncogene (DNA binding)	3	3	9.6%	42674
[View Peptides]	TXNRD1	thioredoxin reductase 1	3	3	9.6%	54707
[View Peptides]	KPNA4	karyopherin alpha 4 (importin alpha 3)	2	2	9.6%	57887
[View Peptides]	MTA2	metastasis associated 1 family, member 2	4	5	9.6%	75023
[View Peptides]	RFC1	replication factor C (activator 1) 1, 145kDa	7	8	9.6%	128254
[View Peptides]	RPS3A	ribosomal protein S3A	2	8	9.5%	29945
[View Peptides]	GALNT2	UDP-N-acetyl-alpha-D-galactosamine:polypeptide N-acetylgalactosaminyltransferase 2 (GalNAc-T2)	3	3	9.5%	64733
[View Peptides]	HSPA4L	heat shock 70kDa protein 4-like	5	6	9.5%	94486
[View Peptides]	GOT1	glutamic-oxaloacetic transaminase 1, soluble (aspartate aminotransferase 1)	2	4	9.4%	46248
[View Peptides]	SMARCB1	SWI/SNF related, matrix associated, actin dependent regulator of chromatin, subfamily b, member 1	2	2	9.4%	44141
[View Peptides]	APTX	aprataxin	2	3	9.3%	40740
[View Peptides]	NLE1	notchless homolog 1 (Drosophila)	3	4	9.3%	53266
[View Peptides]	FSCN1	fascin homolog 1, actin-bundling protein (Strongylocentrotus purpuratus)	3	13	9.3%	54530
[View Peptides]	SNW1	SNW domain containing 1	3	3	9.3%	61495
[View Peptides]	DDX52	DEAD (Asp-Glu-Ala-Asp) box polypeptide 52	3	4	9.3%	67466
[View Peptides]	COPG	coatomer protein complex, subunit gamma	5	5	9.3%	97718
[View Peptides]	SKIV2L2	superkiller viralicidic activity 2-like 2 (S. cerevisiae)	7	12	9.3%	117805
[View Peptides]	NOLA1	nucleolar protein family A, member 1 (H/ACA small nucleolar RNPs)	2	2	9.2%	22348
[View Peptides]	CAPG	capping protein (actin filament), gelsolin-like	2	2	9.2%	38518
[View Peptides]	HNRPLL	heterogeneous nuclear ribonucleoprotein L-like	3	3	9.2%	60083
[View Peptides]	DDB1	damage-specific DNA binding protein 1, 127kDa	7	12	9.2%	126968
[View Peptides]	KPNB1	karyopherin (importin) beta 1	5	11	9.1%	97170
[View Peptides]	ANXA6	annexin A6	4	5	9.1%	75873
[View Peptides]	gi|399184, gi|63...	gi|63102451|gb|AAH95440.1| CAP1 protein [Homo sapiens], gi|5453595|ref|NP_006358.1| adenylyl cyclase...	2	3	9.1%	51673
[View Peptides]	CTH	cystathionase (cystathionine gamma-lyase)	2	3	9.1%	44508
[View Peptides]	TAF15	TAF15 RNA polymerase II, TATA box binding protein (TBP)-associated factor, 68kDa	4	14	9.0%	61830
[View Peptides]	CTTN	cortactin	3	4	8.9%	61636
[View Peptides]	PSMC4	proteasome (prosome, macropain) 26S subunit, ATPase, 4	2	4	8.9%	47366

[View Peptides]	gi|3334339, gi|1...	gi|3334339|sp|Q92747|ARC1A_HUMAN Actin-related protein 2/3 complex subunit 1A (SOP2-like protein), g...	2	2	8.9%	41583
[View Peptides]	CHD4	chromodomain helicase DNA binding protein 4	11	15	8.8%	217989
[View Peptides]	CD2BP2	CD2 (cytoplasmic tail) binding protein 2	2	2	8.8%	37646
[View Peptides]	MDH1	malate dehydrogenase 1, NAD (soluble)	2	3	8.7%	36426
[View Peptides]	TWF1	twinfilin, actin-binding protein, homolog 1 (Drosophila)	2	2	8.7%	42209
[View Peptides]	ARHGAP1	Rho GTPase activating protein 1	2	2	8.7%	50436
[View Peptides]	EXOSC4	exosome component 4	2	2	8.6%	26383
[View Peptides]	HSPH1	heat shock 105kDa/110kDa protein 1	4	5	8.5%	96865
[View Peptides]	LTA4H	leukotriene A4 hydrolase	3	3	8.5%	69285
[View Peptides]	ERCC2	excision repair cross-complementing rodent repair deficiency, complementation group 2 (xeroderma pig...	4	4	8.4%	86909
[View Peptides]	SMC3	structural maintenance of chromosomes 3	6	9	8.4%	141541
[View Peptides]	DNMT1	DNA (cytosine-5-)-methyltransferase 1	8	13	8.4%	183164
[View Peptides]	GRSF1	G-rich RNA sequence binding factor 1	2	4	8.3%	50170
[View Peptides]	EIF3B	eukaryotic translation initiation factor 3, subunit B	4	5	8.2%	92492
[View Peptides]	ERCC3	excision repair cross-complementing rodent repair deficiency, complementation group 3 (xeroderma pig...	4	5	8.2%	89278
[View Peptides]	CIRH1A	cirrhosis, autosomal recessive 1A (cirhin)	3	7	8.2%	76890
[View Peptides]	PSMC1	proteasome (prosome, macropain) 26S subunit, ATPase, 1	2	2	8.2%	49185
[View Peptides]	SERPINB6	serpin peptidase inhibitor, clade B (ovalbumin), member 6	2	2	8.2%	42590
[View Peptides]	FDPS	farnesyl diphosphate synthase (farnesyl pyrophosphate synthetase, dimethylallyltranstransferase, ger...	2	3	8.2%	40532
[View Peptides]	COPS4	COP9 constitutive photomorphogenic homolog subunit 4 (Arabidopsis)	2	2	8.1%	46269
[View Peptides]	SEPT8	septin 8	3	7	8.1%	55756
[View Peptides]	RANBP5	RAN binding protein 5	5	7	8.1%	123630
[View Peptides]	MYH10	myosin, heavy chain 10, non-muscle	9	18	8.0%	228937
[View Peptides]	ABCE1	ATP-binding cassette, sub-family E (OABP), member 1	3	3	8.0%	67314
[View Peptides]	NOLC1	nucleolar and coiled-body phosphoprotein 1	4	6	7.9%	73720
[View Peptides]	AURKA	aurora kinase A	2	2	7.9%	45809
[View Peptides]	PRSS3	protease, serine, 3 (mesotrypsin)	2	3	7.9%	32499
[View Peptides]	RPL13A	ribosomal protein L13a	2	4	7.9%	23577
[View Peptides]	BCKDK	branched chain ketoacid dehydrogenase kinase	2	5	7.8%	46360
[View Peptides]	GANAB	glucosidase, alpha; neutral AB	5	5	7.7%	106874
[View Peptides]	LMNA	lamin A/C	4	6	7.7%	74140
[View Peptides]	STRAP	serine/threonine kinase receptor associated protein	2	3	7.7%	38438
[View Peptides]	VAT1	vesicle amine transport protein 1 homolog (T. californica)	2	2	7.6%	41920
[View Peptides]	RDBP	RD RNA binding protein	2	2	7.6%	43240

[View Peptides]	MAT2A	methionine adenosyltransferase II, alpha	2	3	7.6%	43661
[View Peptides]	RSL1D1	ribosomal L1 domain containing 1	3	3	7.6%	54973
[View Peptides]	ACSL4	acyl-CoA synthetase long-chain family member 4	3	3	7.6%	79188
[View Peptides]	MYBBP1A	MYB binding protein (P160) 1a	5	8	7.6%	148854
[View Peptides]	CPSF1	cleavage and polyadenylation specific factor 1, 160kDa	4	5	7.6%	160883
[View Peptides]	FASN	fatty acid synthase	12	28	7.6%	273397
[View Peptides]	PSMC6	proteasome (prosome, macropain) 26S subunit, ATPase, 6	2	2	7.5%	44173
[View Peptides]	SHMT2	serine hydroxymethyltransferase 2 (mitochondrial)	2	5	7.5%	55993
[View Peptides]	HSPA6	heat shock 70kDa protein 6 (HSP70B')	4	6	7.5%	71028
[View Peptides]	ILF3	interleukin enhancer binding factor 3, 90kDa	4	6	7.5%	95339
[View Peptides]	CTNNA1	catenin (cadherin-associated protein), alpha 1, 102kDa	4	7	7.5%	100071
[View Peptides]	TUBGCP2	tubulin, gamma complex associated protein 2	4	4	7.5%	102534
[View Peptides]	THRAP3	thyroid hormone receptor associated protein 3	7	10	7.5%	108694
[View Peptides]	WDR33	WD repeat domain 33	5	5	7.5%	145921
[View Peptides]	PSMC2	proteasome (prosome, macropain) 26S subunit, ATPase, 2	2	2	7.4%	48634
[View Peptides]	LYAR	Ly1 antibody reactive homolog (mouse)	2	2	7.4%	43615
[View Peptides]	DNM1L	dynamin 1-like	3	4	7.3%	81891
[View Peptides]	SHMT1	serine hydroxymethyltransferase 1 (soluble)	2	2	7.2%	53083
[View Peptides]	SNX9	sorting nexin 9	2	2	7.2%	66592
[View Peptides]	CALD1	caldesmon 1	4	5	7.2%	93250
[View Peptides]	MATR3	matrin 3	3	7	7.2%	94623
[View Peptides]	TLN1	talin 1	9	9	7.2%	269715
[View Peptides]	THOC2	THO complex 2	8	11	7.1%	169581
[View Peptides]	MTA1	metastasis associated 1	3	4	7.1%	80788
[View Peptides]	ANAPC7	anaphase promoting complex subunit 7	3	3	7.1%	63161
[View Peptides]	PSD3, PSMD3	proteasome (prosome, macropain) 26S subunit, non-ATPase, 3, pleckstrin and Sec7 domain containing 3	3	3	7.1%	60978
[View Peptides]	TUFM	Tu translation elongation factor, mitochondrial	2	2	7.1%	49542
[View Peptides]	TERF2IP	telomeric repeat binding factor 2, interacting protein	2	2	7.0%	44260
[View Peptides]	MPHOSPH10	M-phase phosphoprotein 10 (U3 small nucleolar ribonucleoprotein)	2	2	7.0%	78864
[View Peptides]	APEH	N-acylaminoacyl-peptide hydrolase	3	3	7.0%	81225
[View Peptides]	BCLAF1	BCL2-associated transcription factor 1	4	7	7.0%	106122
[View Peptides]	HEATR1	HEAT repeat containing 1	10	16	7.0%	242368
[View Peptides]	DIS3	DIS3 mitotic control homolog (S. cerevisiae)	4	7	6.9%	109003
[View Peptides]	UBTF	upstream binding transcription factor, RNA polymerase I	4	8	6.9%	89406
[View Peptides]	UAP1	UDP-N-acteylglucosamine pyrophosphorylase 1	2	2	6.9%	58769

[View Peptides]	PSMA1	proteasome (prosome, macropain) subunit, alpha type, 1	2	2	6.8%	29556
[View Peptides]	IK	IK cytokine, down-regulator of HLA II	2	4	6.8%	65630
[View Peptides]	ORC3L	origin recognition complex, subunit 3-like (yeast)	3	3	6.8%	82254
[View Peptides]	CLTC	clathrin, heavy chain (Hc)	6	11	6.8%	191613
[View Peptides]	RRP1	ribosomal RNA processing 1 homolog (S. cerevisiae)	2	2	6.7%	52839
[View Peptides]	NMT1	N-myristoyltransferase 1	2	4	6.7%	56806
[View Peptides]	PNN	pinin, desmosome associated protein	3	5	6.7%	81614
[View Peptides]	WDR75	WD repeat domain 75	4	5	6.7%	94499
[View Peptides]	MCM2	minichromosome maintenance complex component 2	3	3	6.7%	101896
[View Peptides]	IPO9	importin 9	4	4	6.7%	115963
[View Peptides]	PRMT1	protein arginine methyltransferase 1	2	2	6.6%	41486
[View Peptides]	ACTR2	ARP2 actin-related protein 2 homolog (yeast)	2	3	6.6%	44761
[View Peptides]	BLMH	bleomycin hydrolase	2	2	6.6%	52562
[View Peptides]	SFRS11	splicing factor, arginine/serine-rich 11	2	6	6.6%	53542
[View Peptides]	CHAF1B	chromatin assembly factor 1, subunit B (p60)	2	2	6.6%	61493
[View Peptides]	STIP1	stress-induced-phosphoprotein 1 (Hsp70/Hsp90-organizing protein)	2	2	6.6%	62639
[View Peptides]	SSRP1	structure specific recognition protein 1	3	4	6.6%	81075
[View Peptides]	TOP3B	topoisomerase (DNA) III beta	3	5	6.6%	96662
[View Peptides]	COPA	coatomer protein complex, subunit alpha	5	5	6.6%	138331
[View Peptides]	HDLBP	high density lipoprotein binding protein (vigilin)	4	4	6.6%	141439
[View Peptides]	BLM	Bloom syndrome	6	6	6.6%	159000
[View Peptides]	NMT2	N-myristoyltransferase 2	2	4	6.6%	56980
[View Peptides]	POLE2	polymerase (DNA directed), epsilon 2 (p59 subunit)	2	3	6.5%	59537
[View Peptides]	ATP5A1	ATP synthase, H+ transporting, mitochondrial F1 complex, alpha subunit 1, cardiac muscle	2	3	6.5%	59751
[View Peptides]	PFKM	phosphofructokinase, muscle	3	4	6.5%	85182
[View Peptides]	RBM25	RNA binding motif protein 25	3	4	6.5%	94122
[View Peptides]	GMIP	GEM interacting protein	4	6	6.5%	106733
[View Peptides]	PAFAH1B1	platelet-activating factor acetylhydrolase, isoform Ib, alpha subunit 45kDa	2	3	6.3%	46638
[View Peptides]	KRT13	keratin 13	2	2	6.3%	49586
[View Peptides]	C6orf182	chromosome 6 open reading frame 182	2	2	6.3%	53649
[View Peptides]	IFIT3	interferon-induced protein with tetratricopeptide repeats 3	2	3	6.3%	55985
[View Peptides]	WDR1	WD repeat domain 1	2	5	6.3%	66194
[View Peptides]	XRCC1	X-ray repair complementing defective repair in Chinese hamster cells 1	3	7	6.3%	69526
[View Peptides]	POR	P450 (cytochrome) oxidoreductase	2	2	6.2%	76690
[View Peptides]	VRK1	vaccinia related kinase 1	2	4	6.1%	45476

[View Peptides]	PRMT5	protein arginine methyltransferase 5	3	4	6.1%	72684
[View Peptides]	RARS	arginyl-tRNA synthetase	2	2	6.1%	75379
[View Peptides]	SMARCC2	SWI/SNF related, matrix associated, actin dependent regulator of chromatin, subfamily c, member 2	4	4	6.1%	132879
[View Peptides]	GATAD2A	GATA zinc finger domain containing 2A	2	2	6.0%	68063
[View Peptides]	SRP72	signal recognition particle 72kDa	2	2	6.0%	74606
[View Peptides]	CPSF2	cleavage and polyadenylation specific factor 2, 100kDa	3	4	6.0%	88487
[View Peptides]	XPO4	exportin 4	4	4	6.0%	130139
[View Peptides]	PPP1R12A	protein phosphatase 1, regulatory (inhibitor) subunit 12A	4	4	5.9%	115281
[View Peptides]	MTA3	metastasis associated 1 family, member 3	2	3	5.9%	67504
[View Peptides]	PACSIN2	protein kinase C and casein kinase substrate in neurons 2	2	2	5.8%	55739
[View Peptides]	TBL3	transducin (beta)-like 3	2	4	5.8%	56047
[View Peptides]	PLK1	polo-like kinase 1 (Drosophila)	2	2	5.8%	68255
[View Peptides]	GTPBP4	GTP binding protein 4	3	3	5.8%	73965
[View Peptides]	MDC1	mediator of DNA damage checkpoint 1	6	6	5.8%	226664
[View Peptides]	EIF5B	eukaryotic translation initiation factor 5B	5	10	5.7%	138799
[View Peptides]	UMPS	uridine monophosphate synthetase (orotate phosphoribosyl transferase and orotidine-5'-decarboxylase)	2	2	5.6%	52222
[View Peptides]	gi|4758644, gi|3...	gi|4758644|ref|NP_004511.1| kinesin heavy chain member 2 [Homo sapiens], gi|3024057|sp|O00139|KIF2_H...	2	3	5.6%	76927
[View Peptides]	MED24	mediator complex subunit 24	3	4	5.5%	110305
[View Peptides]	PFKP	phosphofructokinase, platelet	2	3	5.5%	85596
[View Peptides]	KRT16	keratin 16 (focal non-epidermolytic palmoplantar keratoderma)	2	7	5.5%	51268
[View Peptides]	PPIA	peptidylprolyl isomerase A (cyclophilin A)	2	2	5.5%	18012
[View Peptides]	QPRT	quinolinate phosphoribosyltransferase (nicotinate-nucleotide pyrophosphorylase (carboxylating))	2	2	5.4%	30816
[View Peptides]	SSB	Sjogren syndrome antigen B (autoantigen La)	2	3	5.4%	46837
[View Peptides]	ACSL3	acyl-CoA synthetase long-chain family member 3	2	2	5.4%	80346
[View Peptides]	YARS	tyrosyl-tRNA synthetase	2	2	5.3%	59144
[View Peptides]	ATP6V1A	ATPase, H+ transporting, lysosomal 70kDa, V1 subunit A	2	2	5.3%	68304
[View Peptides]	PDCD6IP	programmed cell death 6 interacting protein	3	3	5.3%	96023
[View Peptides]	ADAR	adenosine deaminase, RNA-specific	3	3	5.3%	135995
[View Peptides]	DSP	desmoplakin	8	11	5.2%	331776
[View Peptides]	IPO7	importin 7	3	4	5.2%	119516
[View Peptides]	MKL1, RBM15	RNA binding motif protein 15, megakaryoblastic leukemia (translocation) 1	3	3	5.2%	107188

[View Peptides]	ABCF1	ATP-binding cassette, sub-family F (GCN20), member 1	5	10	5.2%	95926
[View Peptides]	IDH1	isocitrate dehydrogenase 1 (NADP+), soluble	2	2	5.1%	46659
[View Peptides]	KRT12	keratin 12 (Meesmann corneal dystrophy)	2	5	5.1%	53511
[View Peptides]	CANX	calnexin	2	4	5.1%	67568
[View Peptides]	SMARCC1	SWI/SNF related, matrix associated, actin dependent regulator of chromatin, subfamily c, member 1	4	4	5.1%	122753
[View Peptides]	PABPC1	poly(A) binding protein, cytoplasmic 1	2	3	5.0%	70671
[View Peptides]	PABPC4	poly(A) binding protein, cytoplasmic 4 (inducible form)	2	3	5.0%	70783
[View Peptides]	ZBTB1	zinc finger and BTB domain containing 1	2	4	5.0%	82016
[View Peptides]	ELMO2	engulfment and cell motility 2	2	2	5.0%	82615
[View Peptides]	HMMR	hyaluronan-mediated motility receptor (RHAMM)	2	2	5.0%	84031
[View Peptides]	MYO1C	myosin IC	3	3	5.0%	118038
[View Peptides]	SMC1A	structural maintenance of chromosomes 1A	4	4	5.0%	143233
[View Peptides]	PFAS	phosphoribosylformylglycinamidine synthase (FGAR amidotransferase)	4	4	5.0%	144664
[View Peptides]	KIF14	kinesin family member 14	4	5	5.0%	186490
[View Peptides]	PLS1	plastin 1 (I isoform)	2	3	4.9%	70353
[View Peptides]	CSTF3	cleavage stimulation factor, 3' pre-RNA, subunit 3, 77kDa	2	3	4.9%	82922
[View Peptides]	RRM1	ribonucleotide reductase M1 polypeptide	2	2	4.9%	90070
[View Peptides]	ZNF638	zinc finger protein 638	5	7	4.8%	220623
[View Peptides]	NCBP1	nuclear cap binding protein subunit 1, 80kDa	2	3	4.8%	91839
[View Peptides]	PLS3	plastin 3 (T isoform)	2	3	4.8%	70436
[View Peptides]	RPN1	ribophorin I	2	2	4.8%	68569
[View Peptides]	CAST	calpastatin	2	3	4.7%	76501
[View Peptides]	UHRF1	ubiquitin-like, containing PHD and RING finger domains, 1	2	2	4.7%	89814
[View Peptides]	EPRS	glutamyl-prolyl-tRNA synthetase	4	4	4.7%	163026
[View Peptides]	SNX2	sorting nexin 2	2	2	4.6%	58535
[View Peptides]	NPLOC4	nuclear protein localization 4 homolog (S. cerevisiae)	2	4	4.6%	68120
[View Peptides]	RBM14	RNA binding motif protein 14	2	2	4.6%	69492
[View Peptides]	GAS2L3	growth arrest-specific 2 like 3	2	2	4.6%	75214
[View Peptides]	THOP1	thimet oligopeptidase 1	2	3	4.6%	78840
[View Peptides]	COPB2	coatomer protein complex, subunit beta 2 (beta prime)	2	3	4.6%	102487
[View Peptides]	PRPF6	PRP6 pre-mRNA processing factor 6 homolog (S. cerevisiae)	3	6	4.6%	106925
[View Peptides]	OGT	O-linked N-acetylglucosamine (GlcNAc) transferase (UDP-N-acetylglucosamine:polypeptide-N-acetylgluco...	3	4	4.5%	116924
[View Peptides]	TGM2	transglutaminase 2 (C polypeptide, protein-glutamine-gamma-glutamyltransferase)	2	2	4.5%	77329
[View Peptides]	TUBGCP3	tubulin, gamma complex associated protein 3	2	2	4.4%	103571

[View Peptides]	MCM3	minichromosome maintenance complex component 3	2	3	4.3%	90981
[View Peptides]	MCM8	minichromosome maintenance complex component 8	2	2	4.3%	93697
[View Peptides]	HK1	hexokinase 1	2	2	4.3%	102503
[View Peptides]	POLR1B	polymerase (RNA) I polypeptide B, 128kDa	3	6	4.3%	122138
[View Peptides]	POLR2B	polymerase (RNA) II (DNA directed) polypeptide B, 140kDa	3	4	4.3%	133896
[View Peptides]	KIAA0664	KIAA0664	3	3	4.3%	146669
[View Peptides]	EIF3A	eukaryotic translation initiation factor 3, subunit A	4	4	4.3%	166569
[View Peptides]	TOPBP1	topoisomerase (DNA) II binding protein 1	4	6	4.3%	170677
[View Peptides]	MKI67	antigen identified by monoclonal antibody Ki-67	9	12	4.3%	358747
[View Peptides]	SEC23A	Sec23 homolog A (S. cerevisiae)	2	2	4.2%	86147
[View Peptides]	PPIG	peptidylprolyl isomerase G (cyclophilin G)	2	3	4.2%	88618
[View Peptides]	MED16	mediator complex subunit 16	2	5	4.2%	96793
[View Peptides]	SYMPK	symplekin	3	5	4.2%	141148
[View Peptides]	SIN3A	SIN3 homolog A, transcription regulator (yeast)	3	3	4.2%	145175
[View Peptides]	RTEL1	regulator of telomere elongation helicase 1	3	5	4.1%	152374
[View Peptides]	CDC2L2	cell division cycle 2-like 2 (PITSLRE proteins)	2	4	4.1%	91004
[View Peptides]	PPP1R10	protein phosphatase 1, regulatory (inhibitor) subunit 10	3	5	4.0%	99058
[View Peptides]	SMARCA5	SWI/SNF related, matrix associated, actin dependent regulator of chromatin, subfamily a, member 5	3	3	4.0%	121905
[View Peptides]	CDC2L1	cell division cycle 2-like 1 (PITSLRE proteins)	2	4	4.0%	92707
[View Peptides]	ACIN1	apoptotic chromatin condensation inducer 1	3	5	3.9%	151887
[View Peptides]	MARS	methionyl-tRNA synthetase	2	2	3.9%	101116
[View Peptides]	COPG2	coatomer protein complex, subunit gamma 2	2	2	3.9%	97622
[View Peptides]	WDR43	WD repeat domain 43	2	2	3.8%	79119
[View Peptides]	FGA	fibrinogen alpha chain	2	2	3.8%	94973
[View Peptides]	DNM2	dynamin 2	2	3	3.8%	98064
[View Peptides]	NPEPPS	aminopeptidase puromycin sensitive	2	2	3.8%	103276
[View Peptides]	RIF1	RAP1 interacting factor homolog (yeast)	6	6	3.8%	274464
[View Peptides]	P4HB	procollagen-proline, 2-oxoglutarate 4-dioxygenase (proline 4-hydroxylase), beta polypeptide	2	2	3.7%	57116
[View Peptides]	TRIM25	tripartite motif-containing 25	2	2	3.7%	70990
[View Peptides]	AP3D1	adaptor-related protein complex 3, delta 1 subunit	2	3	3.7%	130158
[View Peptides]	ZMYM3	zinc finger, MYM-type 3	3	3	3.7%	152379
[View Peptides]	MSH6	mutS homolog 6 (E. coli)	3	4	3.7%	152785
[View Peptides]	GCN1L1	GCN1 general control of amino-acid synthesis 1-like 1 (yeast)	6	9	3.7%	292742
[View Peptides]	CUL1	cullin 1	2	2	3.6%	89678
[View Peptides]	LARS	leucyl-tRNA synthetase	2	2	3.6%	134466
[View Peptides]	NCAPD2	non-SMC condensin I complex, subunit D2	2	5	3.6%	157168

[View Peptides]	POLE	polymerase (DNA directed), epsilon	5	11	3.6%	261529
[View Peptides]	CUL4A	cullin 4A	2	2	3.5%	76821
[View Peptides]	MARK3	MAP/microtubule affinity-regulating kinase 3	2	2	3.5%	86944
[View Peptides]	TFIP11	tuftelin interacting protein 11	2	2	3.5%	96820
[View Peptides]	PSMD1	proteasome (prosome, macropain) 26S subunit, non-ATPase, 1	2	2	3.5%	105836
[View Peptides]	XPO1	exportin 1 (CRM1 homolog, yeast)	2	2	3.5%	123386
[View Peptides]	MED1	mediator complex subunit 1	3	3	3.5%	168437
[View Peptides]	PRPF4B	PRP4 pre-mRNA processing factor 4 homolog B (yeast)	2	4	3.4%	116973
[View Peptides]	STAG2	stromal antigen 2	3	3	3.4%	141326
[View Peptides]	FLII	flightless I homolog (Drosophila)	2	2	3.4%	144751
[View Peptides]	MARK2	MAP/microtubule affinity-regulating kinase 2	2	2	3.4%	87911
[View Peptides]	EIF3C	eukaryotic translation initiation factor 3, subunit C	2	3	3.3%	105344
[View Peptides]	YTHDF2	YTH domain family, member 2	2	2	3.3%	62334
[View Peptides]	RBM10	RNA binding motif protein 10	2	2	3.2%	103459
[View Peptides]	MSH2	mutS homolog 2, colon cancer, nonpolyposis type 1 (E. coli)	2	4	3.2%	104743
[View Peptides]	USP7	ubiquitin specific peptidase 7 (herpes virus-associated)	2	3	3.2%	128272
[View Peptides]	CRKRS	Cdc2-related kinase, arginine/serine-rich	3	6	3.2%	164154
[View Peptides]	DCTN1	dynactin 1 (p150, glued homolog, Drosophila)	2	2	3.1%	141694
[View Peptides]	PLCB3	phospholipase C, beta 3 (phosphatidylinositol-specific)	2	3	3.1%	138799
[View Peptides]	VCL	vinculin	2	3	3.1%	123799
[View Peptides]	UPF1	UPF1 regulator of nonsense transcripts homolog (yeast)	2	2	3.0%	124345
[View Peptides]	SEC24C	SEC24 related gene family, member C (S. cerevisiae)	2	2	3.0%	118315
[View Peptides]	GTF3C2	general transcription factor IIIC, polypeptide 2, beta 110kDa	2	2	2.9%	100680
[View Peptides]	SMARCA4	SWI/SNF related, matrix associated, actin dependent regulator of chromatin, subfamily a, member 4	4	5	2.9%	184585
[View Peptides]	MLLT4	myeloid/lymphoid or mixed-lineage leukemia (trithorax homolog, Drosophila); translocated to, 4	3	3	2.9%	205604
[View Peptides]	HCFC1	host cell factor C1 (VP16-accessory protein)	4	4	2.9%	208840
[View Peptides]	CHD7	chromodomain helicase DNA binding protein 7	4	4	2.9%	252482
[View Peptides]	LARP1	La ribonucleoprotein domain family, member 1	2	2	2.7%	123510
[View Peptides]	KIF5B	kinesin family member 5B	2	2	2.6%	109685
[View Peptides]	STAG1	stromal antigen 1	2	2	2.6%	144445
[View Peptides]	POGZ	pogo transposable element with ZNF domain	2	3	2.6%	155269
[View Peptides]	CHD8	chromodomain helicase DNA binding protein 8	3	4	2.6%	230356
[View Peptides]	CAD	carbamoyl-phosphate synthetase 2, aspartate transcarbamylase, and dihydroorotase	3	3	2.6%	242981
[View Peptides]	NIPBL	Nipped-B homolog (Drosophila)	4	6	2.5%	316051
[View Peptides]	SUPT6H	suppressor of Ty 6 homolog (S. cerevisiae)	2	2	2.4%	199071

[View Peptides]	GEMIN5	gem (nuclear organelle) associated protein 5	2	3	2.4%	168560
[View Peptides]	KIF4A	kinesin family member 4A	2	2	2.4%	139881
[View Peptides]	NUMA1	nuclear mitotic apparatus protein 1	3	3	2.3%	238272
[View Peptides]	DYNC1H1	dynein, cytoplasmic 1, heavy chain 1	6	9	2.3%	532412
[View Peptides]	gi|300300, gi|729094	sp|P39880|CUT1_HUMAN CCAAT displacement protein (CDP) (Cut-like 1) , gi|300300|gb|AAB26579.1| CCAAT ...	2	2	2.2%	164353
[View Peptides]	LMO7	LIM domain 7	2	2	2.1%	192708
[View Peptides]	PBRM1	polybromo 1	2	2	2.1%	192946
[View Peptides]	YLPM1	YLP motif containing 1	2	2	2.1%	219983
[View Peptides]	BAT2	HLA-B associated transcript 2	2	2	2.1%	228858
[View Peptides]	PHF3	PHD finger protein 3	2	4	2.1%	229479
[View Peptides]	PDCD11	programmed cell death 11	2	2	2.0%	208733
[View Peptides]	EPPK1	epiplakin 1	5	6	1.8%	553102
[View Peptides]	RRBP1	ribosome binding protein 1 homolog 180kDa (dog)	2	4	1.8%	152472
[View Peptides]	OAS3	2'-5'-oligoadenylate synthetase 3, 100kDa	2	2	1.8%	121165
[View Peptides]	MED13	mediator complex subunit 13	2	2	1.7%	239229
[View Peptides]	NCOR1	nuclear receptor co-repressor 1	2	2	1.7%	270207
[View Peptides]	PCF11	PCF11, cleavage and polyadenylation factor subunit, homolog (S. cerevisiae)	2	2	1.6%	183980
[View Peptides]	NHS	Nance-Horan syndrome (congenital cataracts and dental anomalies)	2	2	1.6%	176699
[View Peptides]	TRRAP	transformation/transcription domain-associated protein	3	3	1.3%	437589
[View Peptides]	AHNAK	AHNAK nucleoprotein	2	2	1.1%	312493
[View Peptides]	PLEC1	plectin 1, intermediate filament binding protein 500kDa	3	4	1.0%	531742
[View Peptides]	CHD6	chromodomain helicase DNA binding protein 6	2	2	0.9%	305412
[View Peptides]	RYR2	ryanodine receptor 2 (cardiac)	2	2	0.8%	564506
[View Peptides]	MDN1	MDN1, midasin homolog (yeast)	2	2	0.6%	632827

YRC Informatics Platform - Version 3.0
Created and Maintained by: Michael Riffle
